# CS-semi5 Inhibits NF-κB Activation to Block Synovial Inflammation, Cartilage Loss and Bone Erosion Associated With Collagen-Induced Arthritis

**DOI:** 10.3389/fphar.2021.655101

**Published:** 2021-07-09

**Authors:** Xiang Li, Xiaonan Tang, Yufei Wang, Changwei Chai, Zhehui Zhao, Haijing Zhang, Ying Peng, Lianqiu Wu

**Affiliations:** Laboratory of Bioactive Substances and Functions of Natural Medicines, Institute of Materia Medica, Chinese Academy of Medical Sciences and Peking Union Medical College, Beijing, China

**Keywords:** rheumatoid arthritis, synovial inflammation, cartilage erosion, bone loss, chondroitin sulfate

## Abstract

Rheumatoid arthritis (RA) is a chronic, systemic autoimmune disease that affects 1% of the population. CS-semi5 is a semisynthetic chondroitin sulfate. In this study, CS-semi5 was shown to have positive effects on a model of collagen-induced arthritis (CIA). CS-semi5 treatment had obvious effects on weight loss and paw swelling in CIA mice. Post-treatment analysis revealed that CS-semi5 alleviated three main pathologies (i.e., synovial inflammation, cartilage erosion and bone loss) in a dose-dependent manner. Further study showed that CS-semi5 could effectively reduce TNF‐α and IL‐1β production in activated macrophages *via* the NF‐κB pathway. CS-semi5 also blocked RANKL-trigged osteoclast differentiation from macrophages. Therefore, CS-semi5 may effectively ameliorate synovial inflammation, cartilage erosion and bone loss in RA through NF-κB deactivation.

## Introduction

Rheumatoid arthritis (RA) is a chronic and systemic autoimmune disease that affects 1% of the population (Firestein, 2003). The typical pathology of RA includes synovial inflammation (Bottini and Firestein, 2013), inflammatory cell infiltration, cartilage destruction and bone erosion (McInnes and Schett, 2011). Currently, a plethora of treatment options focus on pain reduction and remission instead of treating the disease holistically (Singh et al., 2016). However, approved biological therapies are not available for all patients, and these treatments typically lose responsiveness after a short period (Smolen et al., 2017), leading many studies to investigate the prevention of RA development.

Evidence suggests that in RA, both inflammation and joint destruction are involved in macrophages activation (Kinne et al., 2007). RA severity progression is accompanied by aggregation of activated macrophages and secretion of inflammatory cytokines (Mulherin et al., 1996; Tak et al., 1997). Macrophage, other leukocyte subsets or stromal cells all play role in driving RA. Among these cells macrophage may be act as an initiator (Pope, 2002; Schuerwegh et al., 2003; Kim et al., 2005; Alves et al., 2016). In response to the endogenous molecules or the drugs, polarized macrophages including M1 (pro-inflammatory) or M2 (anti-inflammatory) macrophages play a key role in mediating the immune/inflammatory reaction in RA (Tardito et al., 2019). It has been reported that synovial macrophages play a critical role in activating fibroblasts and producing both pro-inflammatory cytokines and destructive enzymes (Li et al., 2012). The relationship between macrophages and RA is mediated by multiple regulators, including mitogen-activated protein kinases (MAPKs) and nuclear factor-κB (NF-κB). The NF-κB pathway can be activated by interleukin-1β (IL-1β), tumor necrosis factor (TNF) and toll-like receptor (TLR) signaling and, in turn, activates transcription of IL-1β, TNF-α, IL-6 and IL-8 (Miagkov et al., 1998). The aforementioned pathways play pivotal roles in mediating inflammatory responses in RA and are extensively involved in RA progression (Yang et al., 2020). Therefore, blockade of NF-κB or MAPKs could be an effective treatment strategy for controlling aggressive inflammation associated with RA.

Chondroitin sulfate (CS) is a type of glycosaminoglycan that attaches to proteins to form proteoglycans (Volpi, 2019). CS can be found in the joint cartilage of animals and is primarily commercially obtained from shark and cow cartilage (Volpi, 2019). Previous research has suggested that CS promotes articular functions and reduces moderate pain (Uebelhart, 2008). However, the homogeneity of natural CS polysaccharides has hindered efforts to precisely characterize their biological functions, and large amounts of these molecules cannot easily be obtained due to the challenges inherent to oligosaccharide synthesis (Clegg et al., 2006). In our previous study, we described a semi-synthesis method capable of more easily generating larger amounts of polysaccharides with less heterogeneity (Yang et al., 2019).

In this paper, we first investigated the therapeutic effect of CS-semi5 on RA by using a bovine type II collagen-induced animal model. Then, we explored the mechanisms by which CS-semi5 inhibits macrophage inflammation to slow the progression of invasion and destruction in RA.

## Materials and Methods

### Synthesis of CS-semi5

CS-semi5 was synthesized in the Institute of Materia Medica, Chinese Academy of Medical Sciences (Yang et al., 2019). To a solution of 26.8 g of CS-A in 400 ml of DMSO was added 40.0 g of Sulphur trioxide trimethyl amine complex at room temperature. The mixture was heated to 60 C and stirred at 60 C for 24 h. The reaction solution was allowed to cool to room temperature, 2000 ml of EtOH was added. The predicated was formed and filtered. The filtered cake was dissolved in 100 ml of water, and adjusted pH to 12.5 ml of EtOH was added, the predicated was formed again, filtered to give crude CS-semi 5 as a brown solid. The crude CS-semi5 was dissolved into 100 ml of water, the solution was dialyzed against distilled water for 2 days and the dialysate was lyophilized to give the CS-semi5 (10.2 g) as off-white powder.

### Animals

Male DBA/1 J mice (18–20 g) were purchased from Beijing HFK Bioscience Co. Inc (Beijing, China, permit No.11400700300231). Animals were kept in controlled conditions (25°C, 60% humidity) with a 12 h light/dark cycle. Experiments were performed in accordance with the Guide for the Care and Use of Laboratory Animals (NIH publication 85–23, revised 1985). All animal procedures were carried out according to the Experimental Animal Welfare and Ethics Committee of the Institute of Materia Medica, Chinese Academy of Medical Sciences, China.

### Reagents and Antibodies

Bovine type II collagen, complete Freund’s adjuvant and incomplete Freund’s adjuvant were purchased from Chondrex (Redmond, WA, United States). Glacial acetic acid and 0.9% sodium chloride were purchased from Sinopharm Chemical Reagent Beijing Co., Ltd (Beijing, China). Formalin was purchased from Beijing Lablead Biotech Co., Ltd., (Beijing, China). Serum separator microtainer tubes were purchased from BioSharp (Hefei, China). Dulbecco’s modified Eagle’s medium (DMEM), phosphate buffered saline (PBS), radio immunoprecipitation assay (RIPA) lysis buffer, protease inhibitor, phosphatase inhibitor and tartrate-resistant acid phosphatasethe (TRAP) staining kit were purchased from Solarbio Science and Technology, Co., Ltd (Beijing, China). Fetal bovine serum (FBS), penicillin and streptomycin were purchased from Gibco Life Technologies (Carlsbad, CA, United States). Enzyme-linked immunosorbent assay (ELISA) kits and recombinant murine receptor activator of nuclear factor-κB ligand (RANKL) were purchased from PeproTech (Rocky Hill, NJ, United States). Lipopolysaccharide (LPS) was purchased from Sigma Chemical Co. (St. Louis, MO, United States). TRIzol was purchased from Invitrogen (Carlsbad, CA, United States). cDNA reverse transcript kit and SYBR green-based quantitative real-time PCR reagents were purchased from Tiangen Biotech Co. Ltd (Beijing, China). Nuclear protein extraction kit was purchased from Nanjing Jiancheng Bioengineering Institute (Nanjing, China). Sodium dodecyl sulfate-polyacrylamide gel electrophoresis (SDS-PAGE) reagents and polyvinylidene fluoride (PVDF) membranes were purchased from Millipore Corp (Bedford, MA, United States). Bovine serum albumin (BSA) was purchased from Roche (Basel, Switzerland). Tris-based saline-Tween 20 (TBST) was purchased from Beijing Applygen Technologies, Inc (Beijing, China). FITC-conjugated secondary antibody, horseradish peroxidase (HRP)-conjugated goat anti-rabbit antibody, and HRP goat anti-mouse secondary antibody were purchased from ZSGB-Bio (Beijing, China). Enhanced chemiluminescence (ECL) reagent was purchased from Tanon (Beijing, China). Triton X-100 was purchased from Sigma-Aldrich (St. Louis, MO, United States). Goat serum and NF-κB-luc were purchased from Beyotime Institute of Biotechnology (Haimen, China). Fluorescent dye 4, 6-diamidino-2-phenylindole dihydrochloride, lipofectamine 2000 reagent and Alexa Fluor-546 rhodamine-phalloidin were purchased from Invitrogen (Carlsbad, CA, United States). Dual luciferase Reporter Assay System was purchased from TransGen (Beijing, China).

Antibodies against IKK, phospho-IKK, IκBα, phospho-IκBα, NFκB p65, and phospho-NF-κB p65 were purchased from Cell Signaling Technology (Beverly, MA, United States). Antibodies against IL‐1β, TNF-α, CD68 and monocyte chemotactic protein 1 (MCP-1) were purchased from Proteintech Group, Inc (Chicago, IL, United States). Antibodies against GAPDH, LaminB1 and β-tubulin were purchased from Abcam (Cambridge, United Kingdom).

### Induction of Collagen-Induced Arthritis Model and CS-semi5 Treatment

**FIGURE 1:**
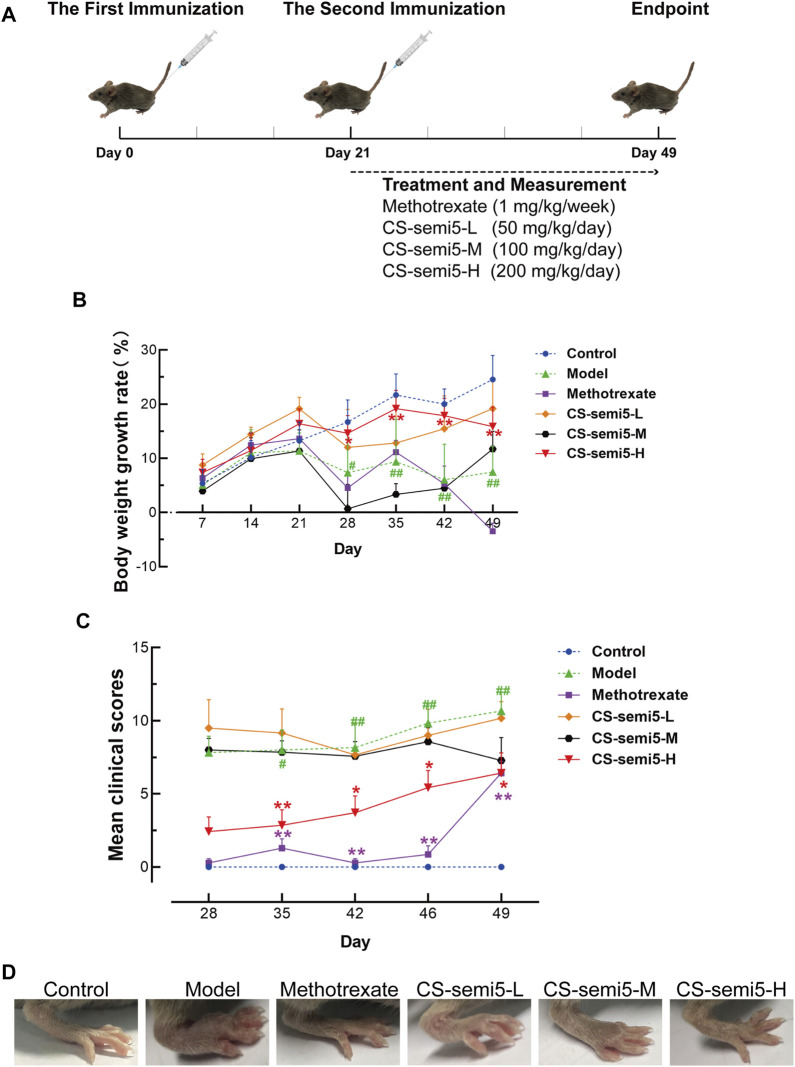
CS-semi5 inhibits the development and progression of collagen-induced arthritis (CIA). **(A)** Schematic diagram of CIA model establishment. **(B)** Body weight growth rate was measured weekly. **(C)** The severity of arthritis swelling was evaluated by mean clinical scores weekly after second immunization. **(D)** Hind paw swelling was photographed on day 49. *n* = 7. ^#^ *p* < 0.05 and ^##^ *p* < 0.01 compared with Control group; *p < 0.05 and **p < 0.01 compared with Model group.

Bovine type II collagen was dissolved in 0.05 M glacial acetic acid at a final concentration of 2 mg/ml overnight at 4°C. The concentration of heat-killed *M. Tuberculosis* H37RA (non-viable) in Complete Freund’s adjuvant was 4 mg/ml. The solution was fully emulsified in an equal volume of complete Freund’s adjuvant with an electric homogenizer in an ice-water bath. Mice were injected subcutaneously with 100 μl emulsion at the base of the tail to induce CIA. The solution was made with incomplete Freund’s adjuvant at a final concentration of 2 mg/ml overnight at 4°C. The same volume of emulsion was injected in the same way on day 21 after the first injection (Figure 1A).

Mice were randomly divided into six groups with seven mice per group: Control group, Model group, Methotrexate group, three doses of CS-semi5 (50 mg/kg, 100 mg/kg and 200 mg/kg, i.e., CS-semi5-L group, CS-semi5-M group and CS-semi5-H group). Mice in the Control group were normal mice only treated with 0.9% sodium chloride *via* intragastric administration. Except Control group, all mice were induced with Bovine type II collagen. Mice in the Model group were only induced Bovine type II collagen and orally daily treated with 0.9% sodium chloride. Mice in the Methotrexate group were intragastrically administrated with 1 mg/kg methotrexate weekly for 28 days following the second immunization. CS-semi5 was dissolved in 0.9% sodium chloride and administered orally (50 mg/kg, 100 mg/kg and 200 mg/kg, respectively) daily for 28 days following the second immunization.

Following day 21 after booster immunization, clinical arthritis was assessed by two independent examiners blinded to experiments according to the following criteria: grade 0, normal; grade 1, mild, but definite redness and swelling of the ankle or wrist, or apparent redness and swelling limited to individual digits, regardless of the number of affected digits; grade 2, moderate redness and swelling of ankle or wrist; grade 3, severe redness and swelling of the entire paw including digits; grade 4, maximally inflamed limb with involvement of multiple joints (Sumariwalla et al., 2002). Each paw was graded from 0 to 4, and the maximum possible score for each mouse was 16. On day 49 after the second immunization, mice were sacrificed. Ankle joints, hind paws and surrounding tissues were obtained from all mice and fixed in 10% formalin. Mouse serum was obtained *via* cardiac puncture and centrifugated in serum separator microtainer tubes.

### Micro-Computed Tomography Analysis

Micro-CT (Siemens Inveon, Germany) scans of the right hind paws were performed at the Institute of Laboratory Animal Science, Chinese Academy of Medical Sciences, Beijing (China). Inveon Research Workplace III software (Germany) was used to reconstruct cross-sectional images into a three-dimensional-structure and to quantitatively determine the degree of bone loss based on bone mineral density (BMD, mg/cm^3^). Two millimeters below the center of the epiphyseal line with 100 slices was selected as the region of interest (ROI) for the analysis.

### Histopathological Examination

Hind paws fixed in formalin were decalcified for two months and embedded in paraffin blocks. Paraffin tissue sections (5 µm thick) were subjected to hematoxylin and eosin (H&E) staining, Safranin O/fast green staining, and toluidine blue and tartrate-resistant acid phosphatase (TRAP) staining.

Samples subjected to H&E staining were analyzed using light microscope (IX71, Olympus) to assess pathological changes of the ankle joints. Histologic scores were evaluated on the basis of synovial inflammation, cartilage destruction and bone erosion (Li et al., 2013a). The histologic score was determined by two independent examiners, in accordance with published standards: 1) inflammatory cell infiltration: score 0, no inflammatory cell infiltration; score 1, mild infiltration; score 2, moderate infiltration; score 3, severe infiltration. 2) synovial hyperplasia: score 0, no hyperplasia; score 1, mild synovial hyperplasia; score 2, moderate synovial hyperplasia; score 3, severe synovial hyperplasia. 3) destruction of articular cartilage: score 0, no destruction; score 1, mild cartilage destruction; score 2, moderate cartilage destruction; score 3, severe cartilage destruction accompanied with loss or crush of the cartilage. 4) destruction of bone: score 0, no destruction; score 1, mild destruction; score 2, moderate destruction; score 3, severe destruction and large areas of bone loss. The total score of each mouse was calculated. The number of synovial lining layers was counted to provide a semi-quantitative assessment of the degree of three features assessed (from 0, absent to 3, strong): enlargement of lining cell layer, cellular density of synovial stroma, leukocytic infiltrate, and then total score was summed up (Chaplan et al., 1994).

Samples subjected to Safranin-O/Fast-green staining were analyzed to evaluate cartilage loss. An Osteoarthritis Research Society International (OARSI) score was assigned to each Safranin-O/Fast-green-stained section according to the OARSI score system: 0 Normal; 0.5 Loss of Safranin-O without structural changes; 1 Small fibrillations without loss of cartilage; 2 Vertical clefts down to the layer immediately below the superficial layer and some loss of surface lamina; 3 Vertical clefts/erosion to the calcified cartilage extending to <25% of the articular surface; 4 Vertical clefts/erosion to the calcified cartilage extending to 25–50% of the articular surface; 5 Vertical clefts/erosion to the calcified cartilage extending to 50–75% of the articular surface; 6 Vertical clefts/erosion to the calcified cartilage extending >75% of the articular surface (Glasson et al., 2010). Articular cartilage areas were dyed red and quantified by tracing regions positive for Safranin-O staining with Image J Software (NIH, United States) (Little et al., 2009).

Samples subjected to TRAP staining were observed, and a light microscope was used to identify osteoclasts. TRAP-positive regions were dyed red and quantitatively analyzed using Image J Software (NIH, United States) (Li et al., 2013b).

### Immunohistochemical Assays

Joint samples were incubated with specific antibodies for murine CD68, NF-κB p65 and MCP-1 according to standard immunohistochemical protocols. Pathological changes were dyed brown. Pathological changes detected by immunohistochemistry were analyzed quantitatively using Image J Software (NIH, United States).

### Cell Culture

The cell line (RAW264.7) was purchased from American Type Culture Collection (ATCC, Manassas, VA, United States) and cultured in DMEM supplemented with 10% (vol/vol) FBS at 37°C under 5% CO_2_ and 95% humidity. Cells were used between passage 3 and 5.

### *In vitro* Osteoclast Genesis Assays

To generate osteoclasts, RAW264.7 cells (1×10^4^ cells/well into 96-well plate) were cultured with 50 ng/ml of RANKL and treated with CS-semi5 at a concentration of 1 μmol/L. After 7 days, cells were fixed in 4% paraformaldehyde for 10 min, and TRAP staining was carried out for osteoclast detection according to the manufacturer’s instructions.

### Enzyme-Linked Immunosorbent Assay

RAW264.7 cells were seeded into 96-well culture plates at 4×10^4^ cells/well. After overnight culture, cells were incubated with LPS (1 μg/ml) and CS-semi5 (0.01, 0.1 and 1 μmol/L) for 24 h. Supernatant was subsequently collected for ELISA analysis.

Concentrations of TNF-α and IL-1β were measured using ELISA kits according to the manufacturer’s instructions. Standard curves were generated based on non-linear regression analysis of known concentrations of TNF-α and IL-1β (Prism, GraphPad Inc., San Diego, CA, United States).

### Western Blot Analysis

RAW264.7 cells were seeded into a 6-well culture plate at 4×10^5^ cells/well. Cells were incubated as described in 2.9 and lysis to extract protein. Nuclear proteins were extracted according to the manufacturer’s instructions for detection of NF-κB p65.

Proteins were subjected to 10% SDS-PAGE and transferred to a PVDF membrane after separation. After being blocked with 5% BSA in TBST at room temperature for 1 h, membranes were incubated with primary antibodies against TNF-α, mature IL-1β, IKK, *p*-IKK, IκBα, *p*-IκBα, NF‐κB p65, p-NF‐κB p65, β-tubulin, GAPDH and LaminB1 overnight at 4°C. Membranes were washed with TBST and then incubated with secondary antibodies at room temperature for 1 h. Membranes were washed with TBST again, and ECL signal was detected using the Tanon 2000 Imaging system (Beijing, China). Blot images were quantified by densitometry using ImageJ Software (NIH, United States).

### Immunofluorescence

RAW264.7 cells were seeded at 5×10^4^ cells/well on glass coverslips in plates and fixed using 4% paraformaldehyde for 30 min at room temperature. Fixed cells were permeabilized for 1 h using 0.2% triton X-100 in PBS containing 10% goat serum, then stained with NF-κB p65 antibody (diluted 1:400) at overnight 4°C. Cells were then washed with PBS and incubated with FITC-conjugated second antibody for 1 h at room temperature. After being washed in PBS, nuclei were counterstained for 3 min with fluorescent dye 4, 6-diamidino-2-phenylindole dihydrochloride. Stained cells were analyzed using confocal laser scanning fluorescence microscopy (Leica TCS SP5-II).

### Luciferase Assay

After transfection with NF-κB and Renilla luciferase reporters into 293T cells, CS-semi5 (0.01, 0.1, and 1 μM) was added to complete DMEM medium containing 1 μg/ml of LPS for 24 h. The double luciferase reporter assay was performed according to the manufacturer’s protocol.

### Statistical Analysis

GraphPad Prism version 8.0.2 was used to calculate the significance among groups. Data were presented as mean ± standard error of mean (SEM). Data were analyzed using one-way analysis of variance followed by Tukey’s HSD post-hoc test. A *p*-value <0.05 was considered as statistically significant difference.

## Results

### CS-semi5 Suppresses Collagen-Induced Weight Loss and Lowers Arthritis Scores

In this study, we used an animal model of CIA to evaluate the therapeutic effects of CS-semi5 treatment. The scheme for collagen-induced arthritis is shown in Figure 1A. Treatment and measurement started on day 21 following the second immunization and continued until day 49 (Figure 1A). Pathological features associated with the CIA induction, such as reduced body weight and increased clinical scores, were apparent and observed to worsen over time in mice in the Model group after the second immunization (Figures 1B,C). As shown in Figure 1B, treatment with CS-semi5 (200 mg/kg once daily) notably decelerated body weight loss of mice from day 28 to day 49. A similar reduction in clinical scores was observed for CS-semi5-H and Methotrexate (Figure 1C). As shown in Figure 1D, hind paw swelling was commonly observed for mice in the Model group at the terminus of the experiment. In contrast, mice in the CS-semi5 (50 mg/kg and 100 mg/kg) groups showed a dose-dependent alleviation of hind paw swelling. Similar changes were observed for mice in the Methotrexate group (Figure 1D). These results suggest that CS-semi5 effectively suppresses the progression of CIA.

### CS-semi5 Limits Synovial Inflammation in CIA Mice

**FIGURE 2:**
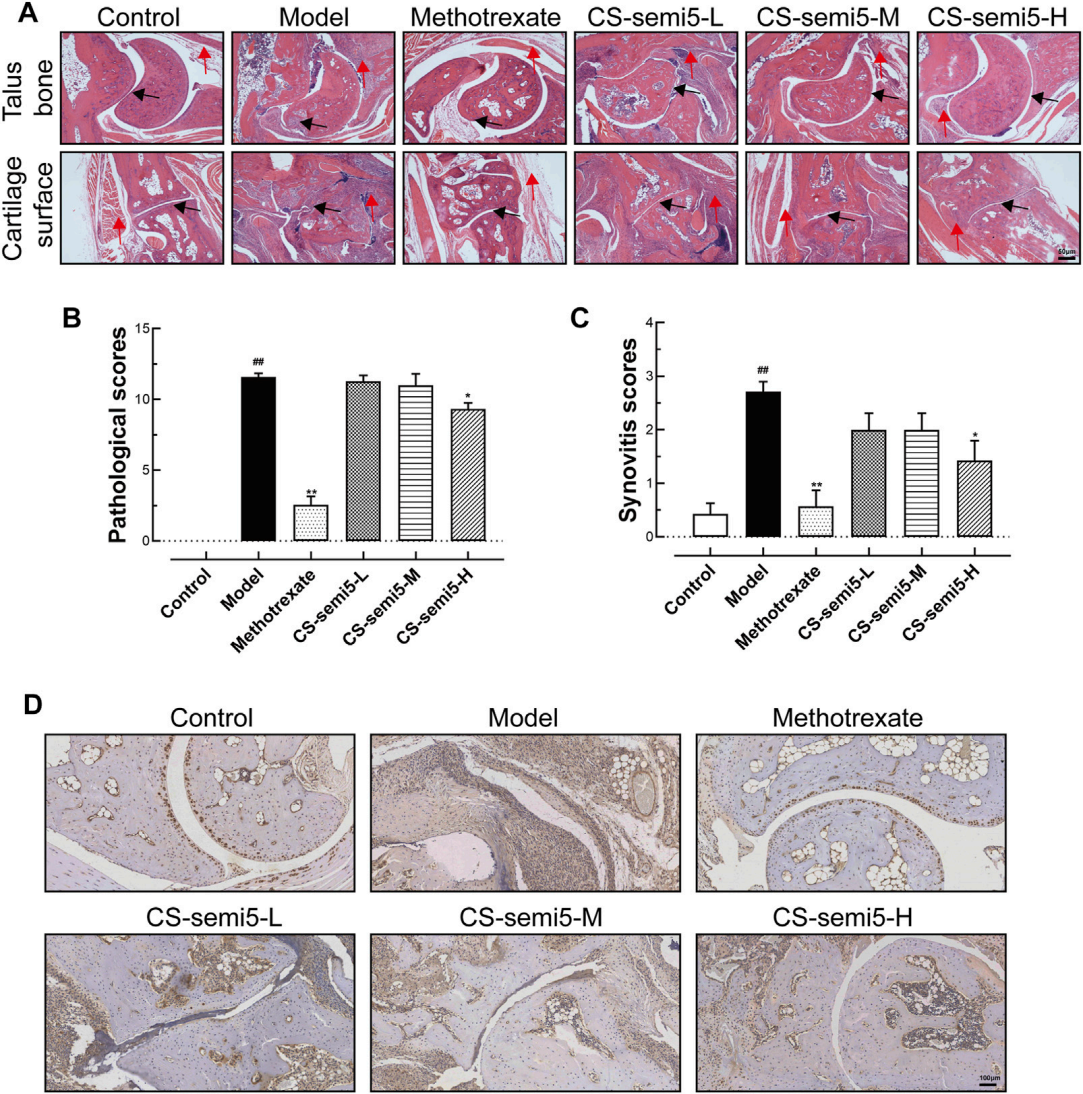
CS-semi5 limits collagen-induced synovium inflammation and inflammatory infiltration in CIA mice. **(A)** Histologic images of talus bone and cartilage surface of ankle joints stained with H&E (40×magnification). Red arrows indicate synovial lining hyperplasia, inflammatory infiltration and pannus formation, and black arrows indicate invasive phenotype of the synovium. Bar indicates 50 μm. **(B)** Pathological scores were calculated. **(C)** The degree of synovitis was semi-quantified based on the synovial lining layers. **(D)** Representative histologic sections of ankle joints following immunostaining with CD68. CD68^+^ macrophages stained in dyed brown. Scale bar represents 100 μm *n* = 7. ^##^ *p* < 0.01 compared with Control group; *p < 0.05 and **p < 0.01 compared with Model group.

To investigate the effects of CS-semi5 on CIA mice, we performed histopathological analysis of joints. It has been established that synovial inflammation and cartilage erosion are critical pathological features of RA (Bottini and Firestein, 2013). Neither synovial inflammation nor cartilage loss was observed in normal mice (Figure 2A). RA-associated histopathological features were observed in Model group, including synovial lining hyperplasia, inflammatory infiltration and pannus formation (indicated by red arrows). Black arrows indicate invasive synovial phenotype, cartilage fracture and cartilage loss observed in CIA mice. In contrast to Model group, mice in the CS-semi5-L and CS-semi5-M groups showed a moderate reduction of inflammation, and mice in the Methotrexate and CS-semi5-H groups showed even more striking changes.

Pathological scores confirmed the presence of severe joint erosion in Model group (Figure 2B). Pathological scores indicated that CS-semi5 treatment (200 mg/kg) markedly inhibited joint destruction and bone erosion. Prominent synovial hyperplasia was analyzed *via* synovitis score (Figure 2C). Immunohistochemical assay showed increased CD68^+^ expression in Model mice compared to normal mice (Figure 2D), indicating a greater number of recruited macrophages. The results showed that CS-semi5 dose-dependently inhibited macrophage infiltration in joint and synovium compared to CIA mice, indicating a more anti-inflammatory microenvironment in mice of CS-semi5 treatment. Collagen induced an increase in the thickness of synovial lining cells, whereas CS-semi5 treatment (200 mg/kg) alleviated both the thickness of these lining cells and synovial inflammation. These data suggest that CS-semi5 effectively relieves synovial injury and joint inflammation in CIA mice.

### CS-semi5 Prevents Cartilage Erosion in CIA Mice

**FIGURE 3:**
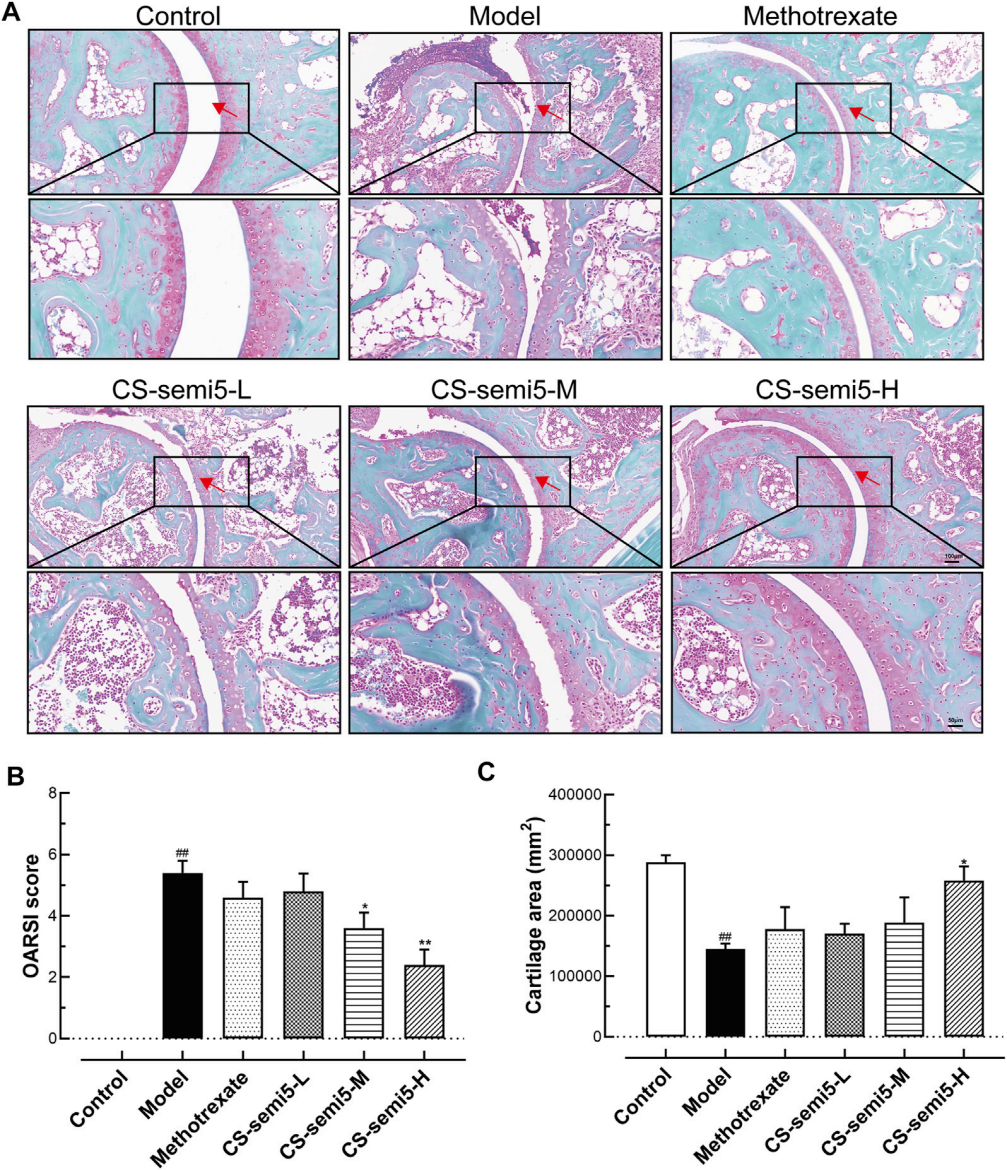
CS-semi5 alleviates collagen-induced cartilage thickness reduction and cartilage loss in CIA mice. **(A)** Safranin O/fast green staining of ankle joints. Red arrows indicate cartilage. Original magnification was 100×, and 200× magnification was used in high-power views. Bar respectively indicates 100 and 50 μm. **(B)** The severity of cartilage loss was analyzed based on the OARSI score system. **(C)** Articular cartilage areas were quantified by tracing Safranin O-positive stained areas. *n* = 7. ^##^ *p* < 0.01 compared with Control group; *p < 0.05 and **p < 0.01 compared with Model group.

Because cartilage erosion is a characteristic feature of RA (Zeng et al., 2016), we examined the capacity of CS-semi5 with respect to cartilage repair. As expected, both cartilage loss and reduced cartilage thickness were observed in Model group, with hypertrophic chondrocytes in the deep cartilage zone also observed in high-power views of Safranin-O/Fast-green sections (Figure 3A). The administration of CS-semi5 significantly ameliorated cartilage degradation in a dose-dependent manner, whereas methotrexate did not increase cartilage thickness. Cartilage degradation was notable in mice in the Model group, as evidenced by markedly increased OARSI scores and reduced cartilage area. However, CS-semi5 dose-dependently limited the progression of cartilage erosion characteristic of the CIA (Figures 3B,C). These results demonstrate that CS-semi5 can limit the development of cartilage loss and repair cartilage erosion.

### CS-semi5 Attenuates Bone Loss and Destruction Induced by CIA

**FIGURE 4:**
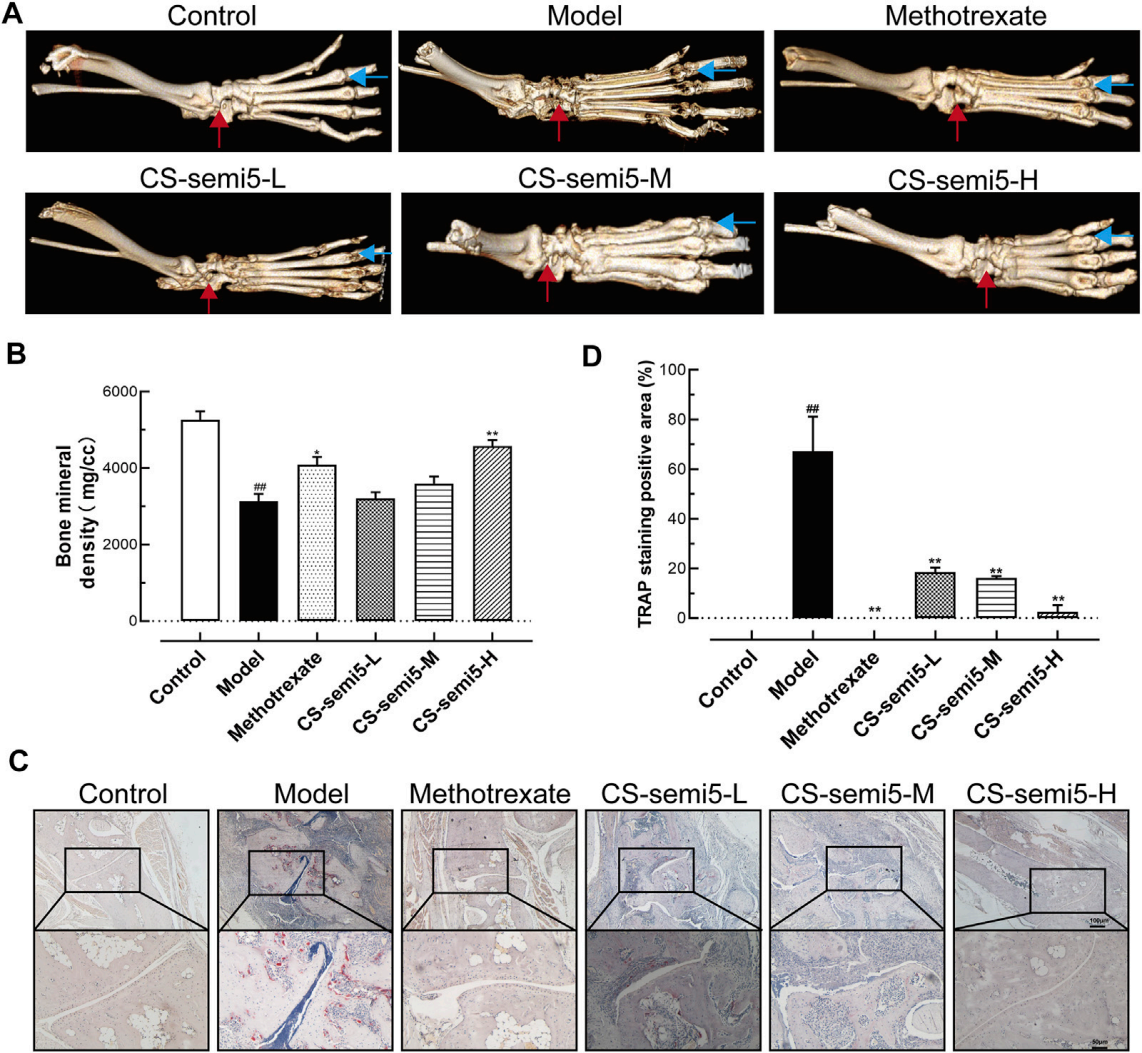
CS-semi5 attenuates bone destruction and bone marrow erosion in CIA mice. **(A)** Representative micro-CT 3D images of hind paws as recorded on day 49. Red arrows indicate the ankle joint structure, and blue arrows indicate the joint space. **(B)** Bone marrow erosion was measured *via* digital quantification of bone mineral density. **(C)** Osteoclasts of the ankle joints were stained with TRAP. Red areas indicate osteoclasts. Original magnification was 40×, and 100× magnification was used in high-power views. Bar respectively indicates 100 and 50 μm. **(D)** TRAP-positive staining was measured by digital quantification. *n* = 7. ^##^ *p* < 0.01 compared with Control group; *p < 0.05 and **p < 0.01 compared with Model group.

To further explore the role of CS-semi5 in ameliorating bone destruction, we analyzed bone mineral density Li et al., 2013a. Joint destruction and bone loss were observed in Model group, as indicated by blue and red arrows (Figure 4A). Micro-CT imaging of the hind paws revealed that both methotrexate and CS-semi5 dose-dependently alleviated joint destruction and bone erosion. Digital quantification of bone mineral density further corroborated the therapeutic effects of methotrexate and CS-semi5 (Figure 4B). Other relevant indicators from micro-CT analysis are shown in Supplementary Figure.

In support of CS-semi5’s inhibitory effects on bone erosion, additional evidence was obtained from TRAP staining (Figure 4C). The region of TRAP-positive osteoclasts was thick in Model group. CS-semi5 (50, 100, and 200 mg/kg) attenuated osteoclast numbers in CIA mice in a dose-dependent manner. Digital evaluation of the TRAP-positive areas further confirmed these results (Figure 4D). These data demonstrate CS-semi5 prevents collagen-induced bone destruction and bone marrow erosion in CIA mice.

### CS-semi5 Regulates Inflammatory Mediators in CIA Mice

**FIGURE 5:**
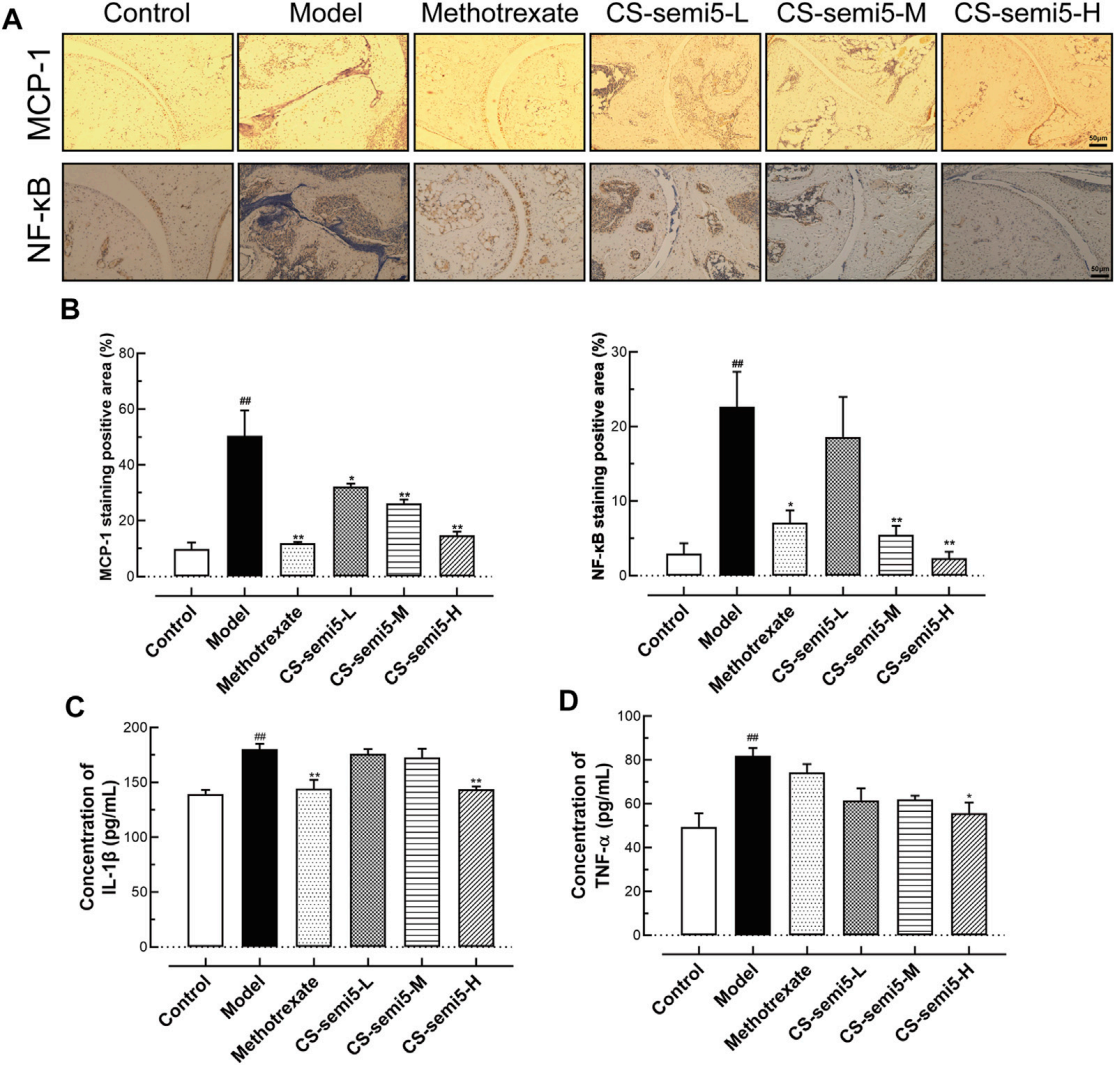
CS-semi5 attenuates CIA-induced arthritis *in vivo.* **(A)** Histologic sections of ankle joints following immunostaining with MCP-1 and NF‐κB antibodies (40×magnification). **(B)** Positive staining was measured by digital quantification. **(C,D)** Serum IL-1β and TNF‐α were assessed by ELISA. *n* = 7. ^##^ *p* < 0.01 compared with Control group; *p < 0.05 and **p < 0.01 compared with Model group.

Inflammatory cytokines play key roles in the pathogenesis of RA (Brennan et al., 1998). To investigate the effect of CS-semi5 on inflammatory mediators and assess its therapeutic efficiency, chemokine (MCP-1) and NF‐κB expression in mice paws were detected *via* immunohistochemical assay. The results showed that CS-semi5 significantly inhibited MCP-1 and NF‐κB expression in CIA mice (Figures 5A,B).

In addition to these cytokines, TNF-α and IL-1β are also considered fundamental, as they are known to induce production of other inflammatory cytokines in RA (Mukaida et al., 1990). Therefore, the effects of CS-semi5 on the expression of proinflammatory cytokines (TNF‐α and IL-1β) in the serum were determined by ELISA. CS-semi5 treatment (200 mg/kg) notably inhibited TNF‐α and IL-1β expression in serum (Figures 5C,D). These data suggest that CS-semi5 reduces inflammation in CIA mice to exert an anti-CIA effect.

### CS-semi5 Suppresses Macrophage Activation and Differentiation *in vitro*

**FIGURE 6:**
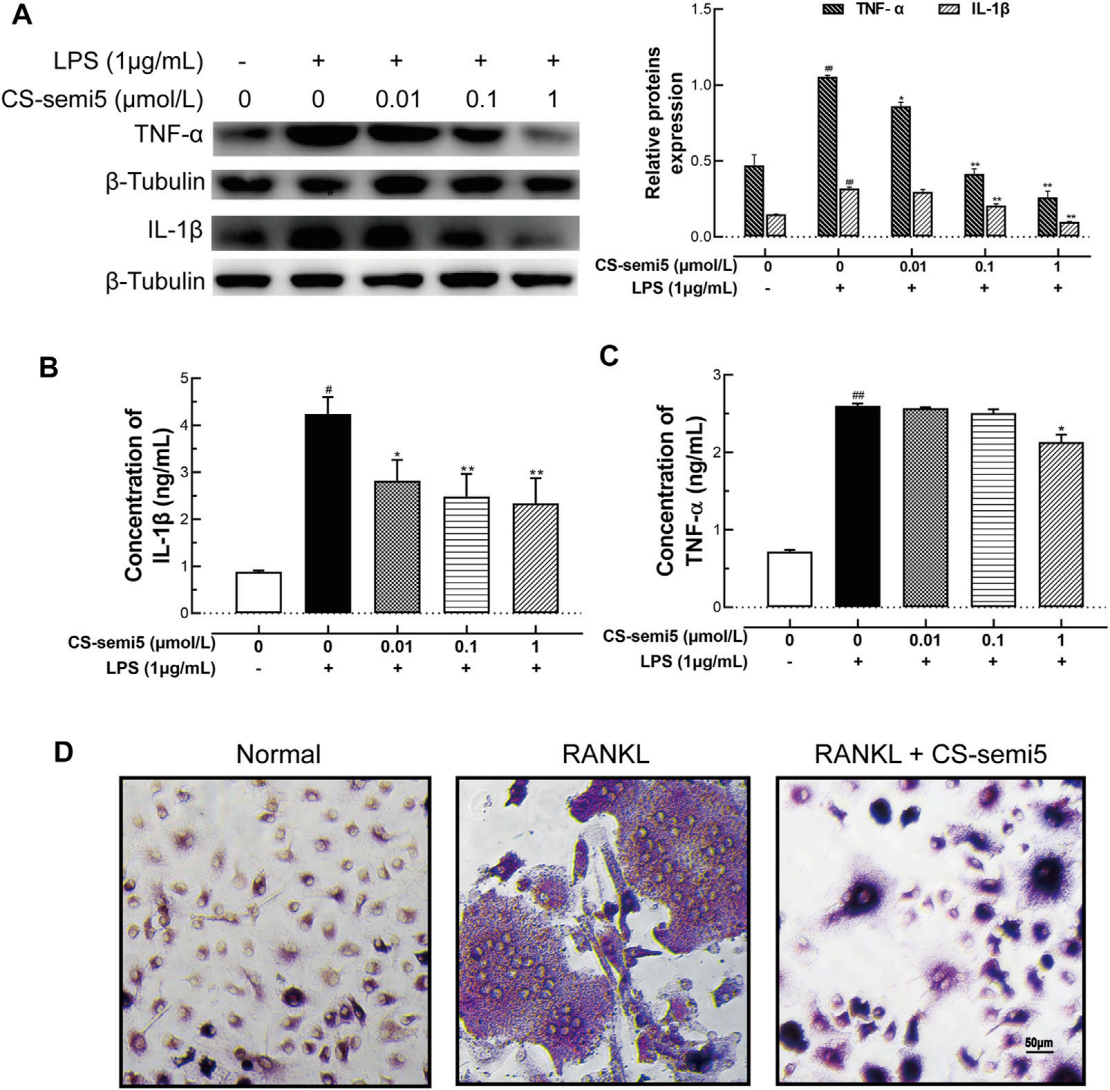
CS-semi5 downregulates TNF‐α and IL‐1β production and blocks osteoclast differentiation in RAW264.7 cells. **(A)** RAW264.7 cells were incubated with or without LPS (1 μg/ml) for 24 h. Secretion of TNF‐α and IL‐1β proteins was evaluated by western blot assay. **(B,C)** Secretion of TNF‐α and IL‐1β proteins was evaluated by ELISA. **(D)** RAW264.7 cells were cultured for 7 days with RANKL (50 ng/ml) or CS-semi5 (1 μmol/ml), then stained for TRAP activity. Bar indicates 50 μm *n* = 3. ^#^ *p* < 0.05 and ^##^ *p* < 0.01 compared with Control Group; *p < 0.05 and **p < 0.01 compared with LPS stimulation Group.

To build on our observations of the effects of CS-semi5 on CIA mice, we next investigated the inhibitory effect *in vitro.* We confirmed *via* Western-blot that CS-semi5 dose-dependently reduced TNF‐α and mature IL-1β protein expression in RAW264.7 (Figure 6A). Consistent with a role in protein inhibition, CS-semi5 treatment decreased mature IL-1β protein secretion in a dose-dependent manner and reduced TNF‐α protein secretion at high concentrations (Figures 6B,C). These data show that CS-semi5 reduces production of proinflammatory cytokines in RAW264.7 cells.

To examine the effects of CS-semi5 on RANKL-induced osteoclast genesis, we incubated RANKL-treated RAW264.7 cells with 1 μmol/L CS-semi5. TRAP-positive cells containing more than three nuclei were counted as osteoclasts. CS-semi5 treatment reduced the number of RANKL-induced osteoclasts (Figure 6D). These data suggest that CS-semi5 inhibits RANKL-induced osteoclast genesis.

### CS-semi5 Inhibits Macrophage Activation *via* the NF‐κB Pathway

**FIGURE 7:**
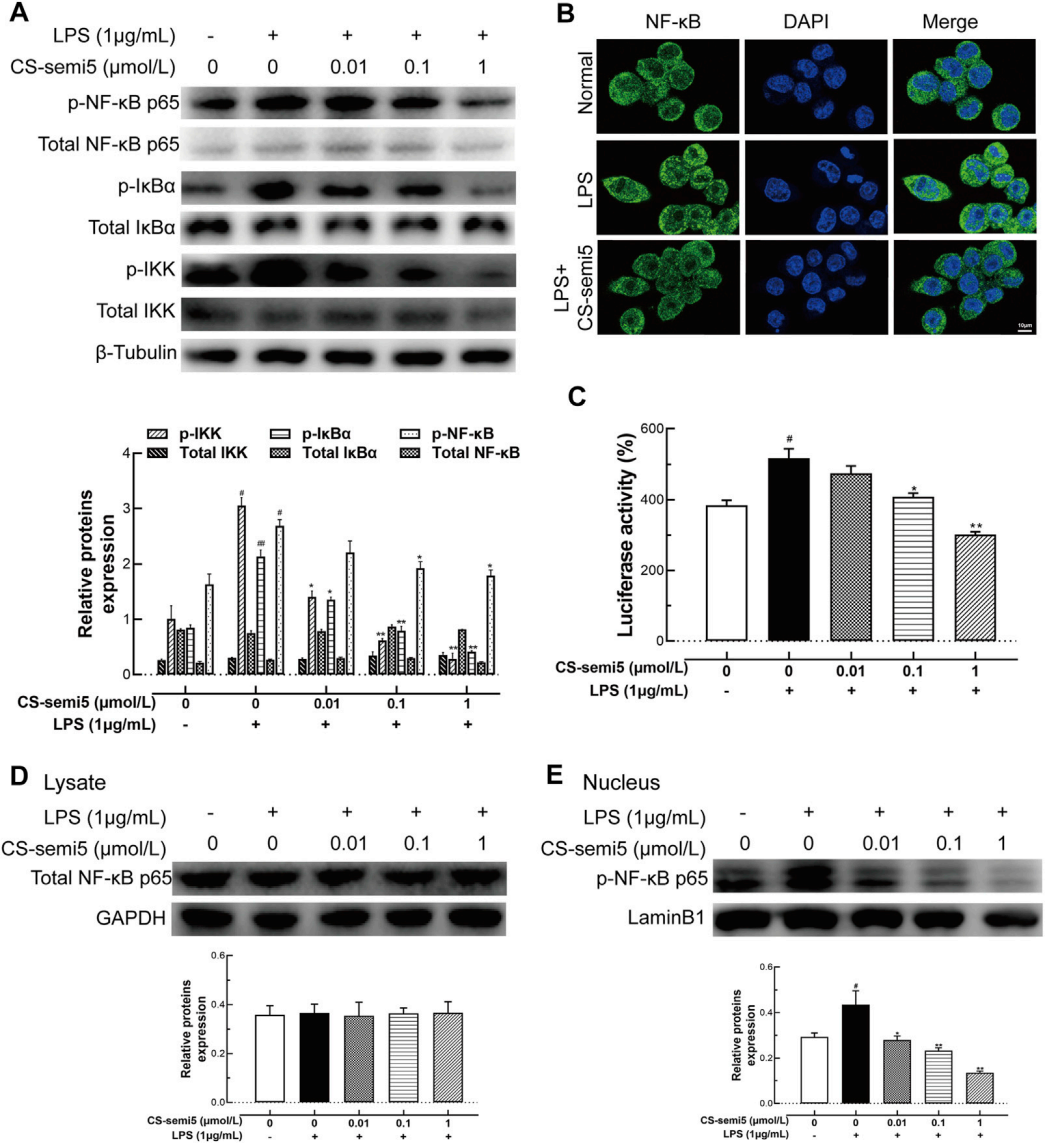
CS-semi5 inhibits activation of the NF‐κB pathway. **(A)** RAW264.7 cells were treated with CS-semi5 (0.01, 0.1 or 1 μmol/L) for 24 h. IKK, IκBα, and NF‐κB phosphorylation was examined by western blot. **(B)** LPS-induced nuclear translocation of NF‐κB p65 was assessed by confocal laser fluorescence microscopy. Cells were immuno-stained with an NF‐κB p65 antibody (FITC, green), and nuclei were stained with DAPI in blue. Bar indicates 10 μm. **(C)** 293T cells were transfected with NF‐κB luciferase plasmid, then treated with CS-semi5 for 24 h. Luciferase activity was assayed. **(D,E)** RAW264.7 cells were stimulated with LPS and treated with CS-semi5 (0.01, 0.1 or 1 μmol/L) for 24 h. NF‐κB p65 in the cell lysis solution and in the nucleus were respectively determined by western blot. *n* = 3. ^#^ *p* < 0.05 and ^##^ *p* < 0.01 compared with Control Group; *p < 0.05 and **p < 0.01 compared with LPS stimulation Group.

Increased NF‐κB activation has been linked to the progression of aggressive inflammation in RA (Makarov, 2001). Therefore, we evaluated the effect of CS-semi5 on NF‐κB activity. CS-semi5 dose-dependently inhibited phosphorylation of IKK, IκBα, and NF‐κB in RAW264.7 cells incubated with LPS (1 μg/ml) (Figure 7A). Furthermore, LPS stimulation increased nuclear translocation of NF‐κB compared to normal control, while CS-semi5 (1 μmol/L) significantly reduced levels of nuclear NF‐κB (Figure 7B). NF‐κB suppression was further confirmed by analyzing NF‐κB luciferase activity (Figure 7C). The production of total NF‐κB p65 in the cell lysate solution was not affected by CS-semi5 treatment (Figure 7D). We also determined that CS-semi5-treated cells were characterized by reduced p65 phosphorylation and nuclear accumulation (Figure 7E). These results demonstrate that, in RAW264.7 cells, CS-semi5 reduces inflammatory disorders *via* the NF‐κB pathway.

## Discussion

RA greatly hinders the ability of many patients to participate in daily activities, including household work, and reduces health-related quality of life, leading to severe disability and mortality (Solomon et al., 2003). The most commonly used medications in RA patients are disease-modifying anti-rheumatic drugs (DMARDs), which are limited by gastrointestinal toxicity. However, DMARDs do not relive cartilage erosion, joint damage or bone loss in RA patients (Lopez-Pedrera et al., 2020). Chondroitin sulfate (CS) and glucosamine are glycosaminoglycans that are considered to be symptomatic slow-acting drugs for osteoarthritis (SYSADOA) (Henrotin et al., 2014). Moreover, CS is major component of cartilage and displays many biological characteristics, such as antitumor, anticoagulant, and anti-inflammatory properties, biocompatibility, and immune-regulatory properties (Lian et al., 2013; Ustyuzhanina et al., 2017). The existence of marine animal sources of CS is compatible with low toxicity and medicinal properties (Wang et al., 2020). In addition, CS has also been reported in clinical trials to show symptomatic efficacy in knee osteoarthritis (Bruyere et al., 2008). It has been shown that CS can improve articular function and ease joint swelling, pain and effusion (Clegg et al., 2006). CS is sold in America as a dietary supplement, whereas it has been registered as a medication in Europe (Messina et al., 2019). In this study, we investigated the effects of CS in an animal model of CIA and explored its potential therapeutic effect on RA. Same with other preclinical studies, we started the administration after the second immunization before RA clinical symptoms completely appeared (Huang et al., 2019; Sun et al., 2019).

**FIGURE 8:**
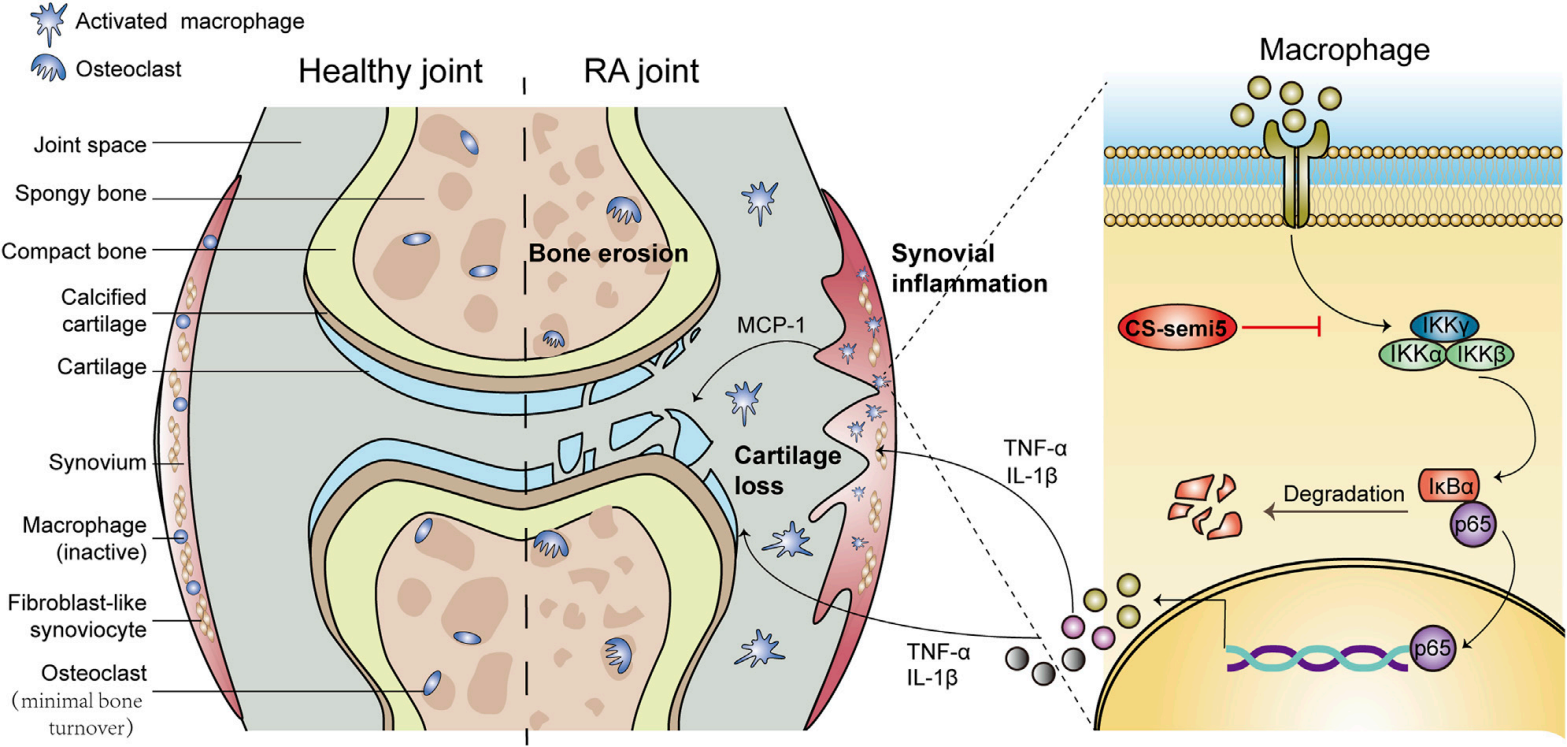
Schematic diagram summarizing the mechanisms by which CS-semi5 alleviates synovial inflammation, cartilage erosion and bone loss in RA. CS-semi5 effectively inhibits the NF-κB signaling pathway, including p65 nuclear translocation and phosphorylation of *p*-IKKα/β and p-NF-κB, both *in vivo* and *in vitro*. CS-semi5 also reduces TNF‐α and IL‐1β production by activated macrophages and blocks RANKL-trigged osteoclast differentiation of macrophages.

It has been established that RA involves a complex interplay between synovial inflammation, cartilage erosion and bone destruction (McInnes and Schett, 2011). In RA, the synovium can acquire aggressive phenotype, including synovial inflammation, synovial hyperplasia and inflammatory cell infiltration; it can also play a critical role in regulating cartilage and bone loss by secreting proinflammatory cytokines (Bottini and Firestein, 2013). Cartilage erosion is also a key trigger of RA processes through the destruction of surface cartilage and joint-space narrowing (McInnes and Schett, 2011). Another notable feature of RA is bone destruction meditated by excessive activation of osteoclasts (Boyle et al., 2003). The activation and invasion of osteoclasts promotes prolonged inflammation and erodes the periosteal surface adjacent to the articular cartilage (Gravallese et al., 1998). Consistent with the above description, CIA mice were characterized by aggressive synovial inflammation, cartilage erosion and bone loss, ultimately leading to weight loss and paw swelling (Figure 8). CS-semi5 treatment, however, was able to effectively limit the development of RA and attenuate weight loss and paw swelling in CIA mice. CS-semi5 dose-dependently alleviated three main RA pathologies (i.e., synovial inflammation, cartilage erosion and bone loss), as shown in Figures 1–4.

Previous reports showed that infiltration of activated macrophages results in cartilage and bone destruction (McInnes and Schett, 2017). In RA, activated macrophages accumulate in the joint capsule and promote synovial inflammation and cartilage erosion by secreting proinflammatory factors (Lee et al., 2017; Wang et al., 2017; Weyand et al., 2017). Chemokine MCP-1, TNF-α and IL‐1β play critical roles in the pathology of RA, ultimately leading to chronic inflammation and joint destruction (McInnes and Schett, 2007; Gaffen et al., 2014; Sucur et al., 2017). In particular, macrophage-produced TNF-α is a primary therapeutic target in RA (Feldmann and Maini, 2008). Macrophages, as osteoclast precursors, can differentiate into osteoclasts in response to receptor activator of nuclear factor (NF)-κB ligand (RANKL) (Walsh et al., 2006). Osteoclasts secrete acid and lytic enzymes that degrade bone matrix, causing bone resorption and lowering bone density (Clegg et al., 2006). Therefore, we sought to investigate whether CS-semi5 was able to alleviate inflammatory action and differentiation in RAW264.7 cells. Consistent with our hypothesis, CS-semi5 exerted anti-RA therapeutic effects *via* the suppression of TNF‐α and IL‐1β production in activated macrophages. In this article, we mainly studied the anti-inflammatory effect of CS-semi5. In future research, we will conduct further research based on other stromal, cartilage and macrophage subpopulations.

NF-κB is a critical transcription factor that is extraordinarily important in inflammation and immunity (Hayden and Ghosh, 2008; Vallabhapurapu and Karin, 2009). NF-κB has also been studied as a key signaling factor involved in the development of synovial inflammation and joint destruction (Miagkov et al., 1998). Previous research has established that in RA, macrophage-produced TNF-α and IL‐1β are completely dependent on NF-κB (Bondeson et al., 1999). Furthermore, NF-κB-null mice failed to generate mature osteoclasts (Franzoso et al., 1997). Receptor activator of nuclear factor (NF)-κB ligand (RANKL) is a major osteoclastogenic molecule. In addition, NF-κB signal is involved in osteoclast activation and survival (Miyazaki et al., 2000). To investigate the mechanism by which CS-semi5 affects regulation of macrophage inflammation and differentiation, we evaluated the effect of CS-semi5 on the NF-κB pathway. To this end, we found that CS-semi5 downregulated LPS-induced phosphorylation of IKKα/β and IκBα, inhibited p65 nuclear translocation and reduced production of TNF‐α and IL‐1β (Figure 7). According to our results in this study and other unpublished results, we think CS-semi5 is a multi-targets compound and has effect on NF-κB and monocytes. In future, more experiments will be needed to elucidate the detailed mechanism how CS-semi5 inhibits NF-κB or plays its role by connecting M1 or M2 macrophages and monocytes together (Chou et al., 2005; Gopalakrishnan et al., 2008; Henrotin and Lambert, 2013).

In this study, CS-semi5 and Methotrexate administration were started after the second immunization before RA clinical symptoms completely appeared, which was in accord with many other researches. But because there are still no signs of inflammation at day 21 in CIA model, true therapeutic effect of CS-semi5 on RA would need more experiments to be assessed.

Collectively, CS-semi5 greatly limited synovial inflammation and ameliorated cartilage erosion and bone loss *in vivo* in CIA mice (Figure 8). CS-semi5 reduced TNF‐α and IL‐1β production by activated macrophages. CS-semi5 also effectively inhibited the NF-κB signaling pathway, including p65 nuclear translocation and phosphorylation of *p*-IKKα/β and p-NF-κB, both *in vivo* and *in vitro*.

## Conclusion

In summary, CS-semi5 was demonstrated to have positive effects on RA *via* the NF-κB pathway. CS-semi5 has development potential as a promising candidate for the treatment of RA.

## Data Availability

The raw data supporting the conclusion of this article will be made available by the authors, without undue reservation.
