# Supplementary figures and images for: CS-semi5 Inhibits NF-κB Activation to Block Synovial Inflammation, Cartilage Loss and Bone Erosion Associated With Collagen-Induced Arthritis
Source: Front Pharmacol. 2021 Jul 9;12:655101. doi: 10.3389/fphar.2021.655101 (PMC8298759; doi:10.3389/fphar.2021.655101)

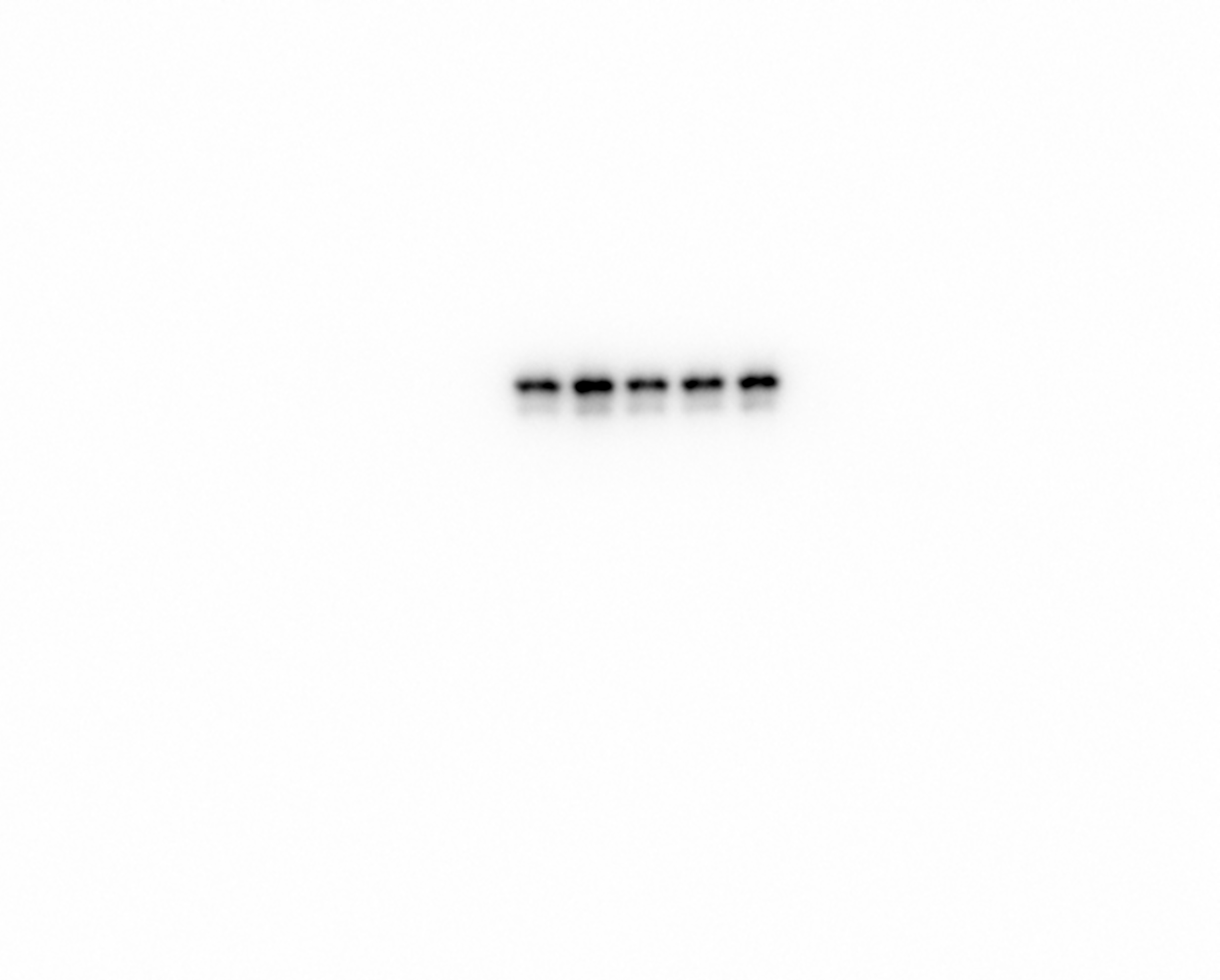

Supplement: Supplementary file 1 [file DataSheet1.ZIP › whole images of WB/IKBa.Tif]

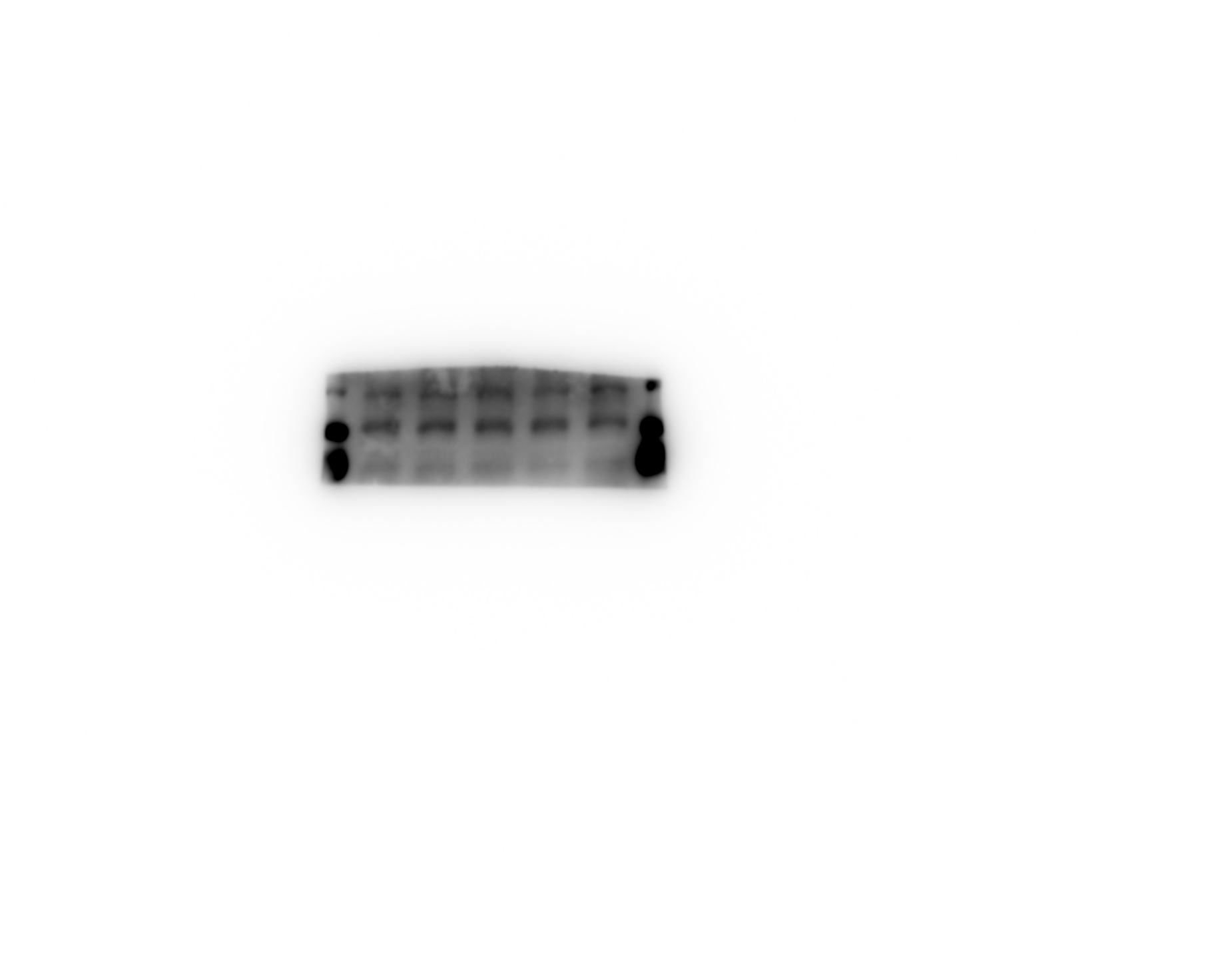

Supplement: Supplementary file 1 [file DataSheet1.ZIP › whole images of WB/IKK.Tif]

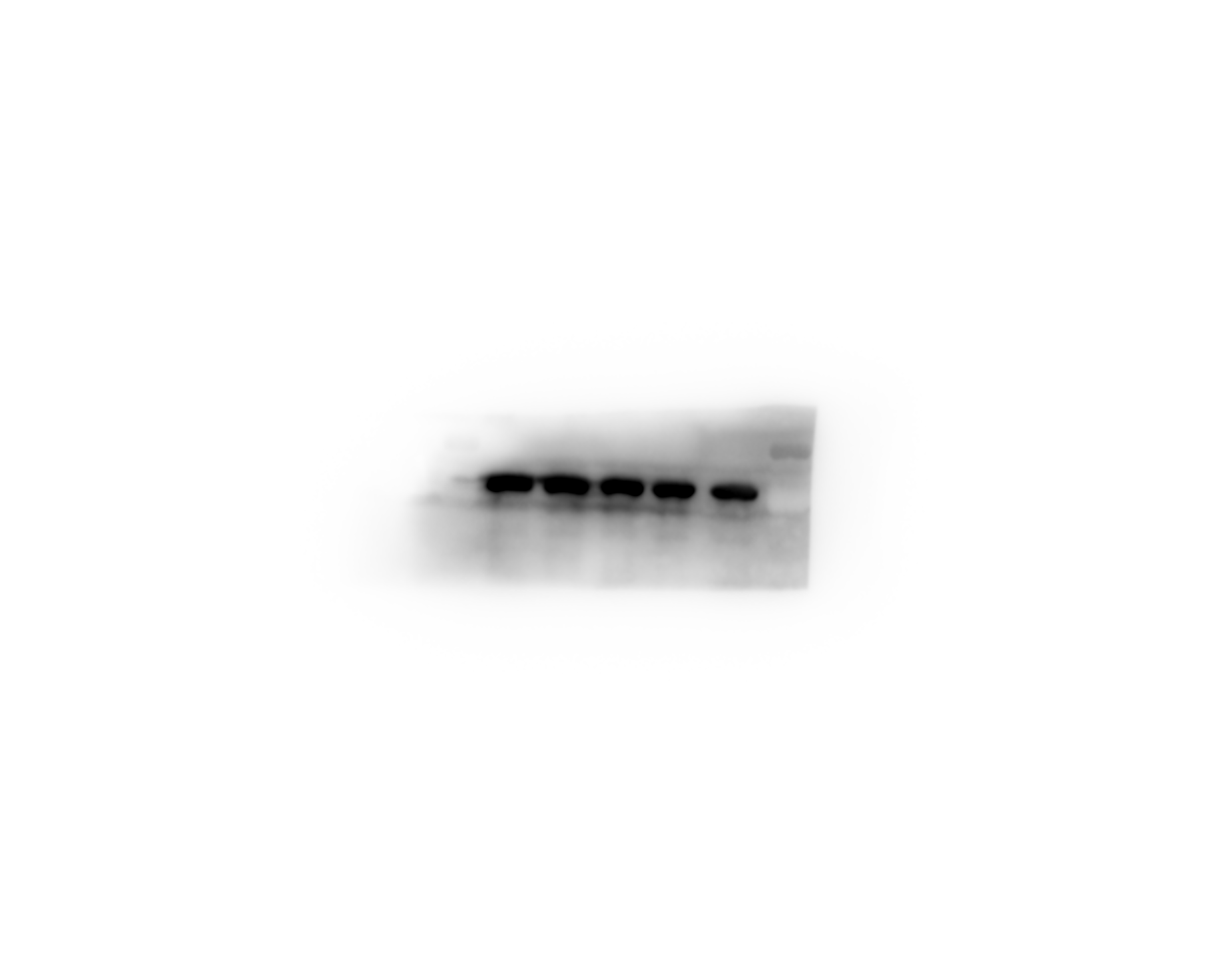

Supplement: Supplementary file 1 [file DataSheet1.ZIP › whole images of WB/LaminB1.Tif]

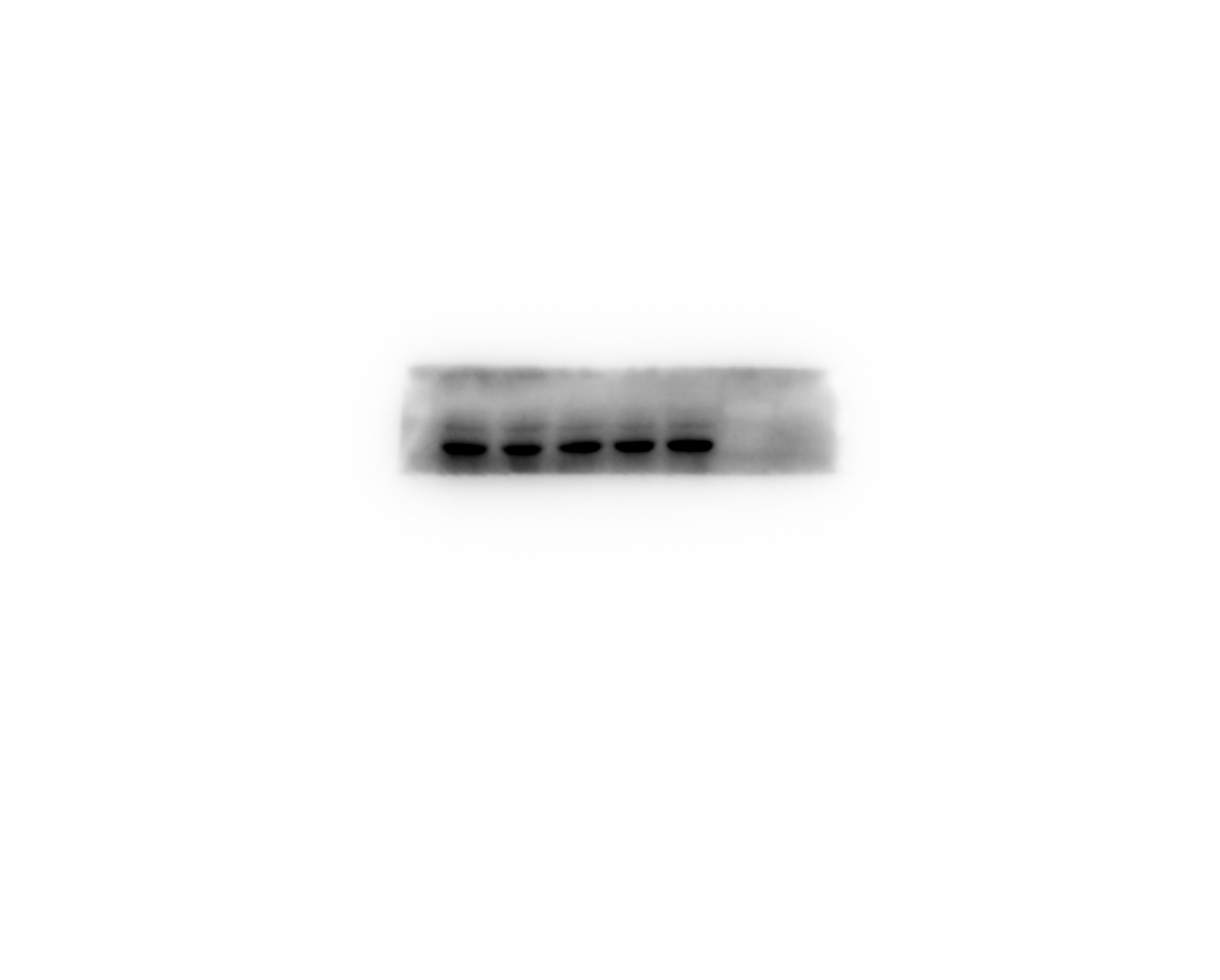

Supplement: Supplementary file 1 [file DataSheet1.ZIP › whole images of WB/Lysate GAPDH.Tif]

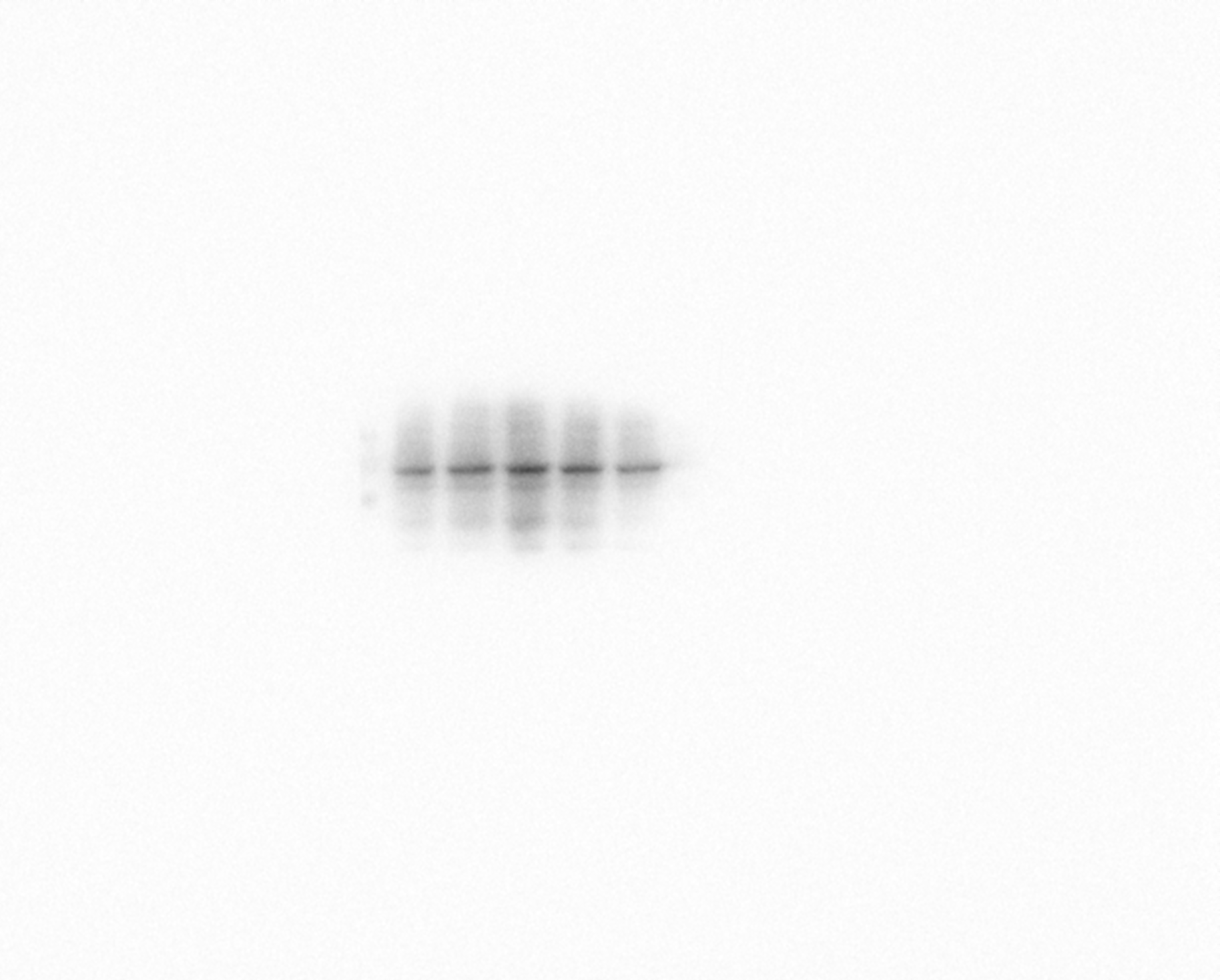

Supplement: Supplementary file 1 [file DataSheet1.ZIP › whole images of WB/NF-KB-P65.Tif]

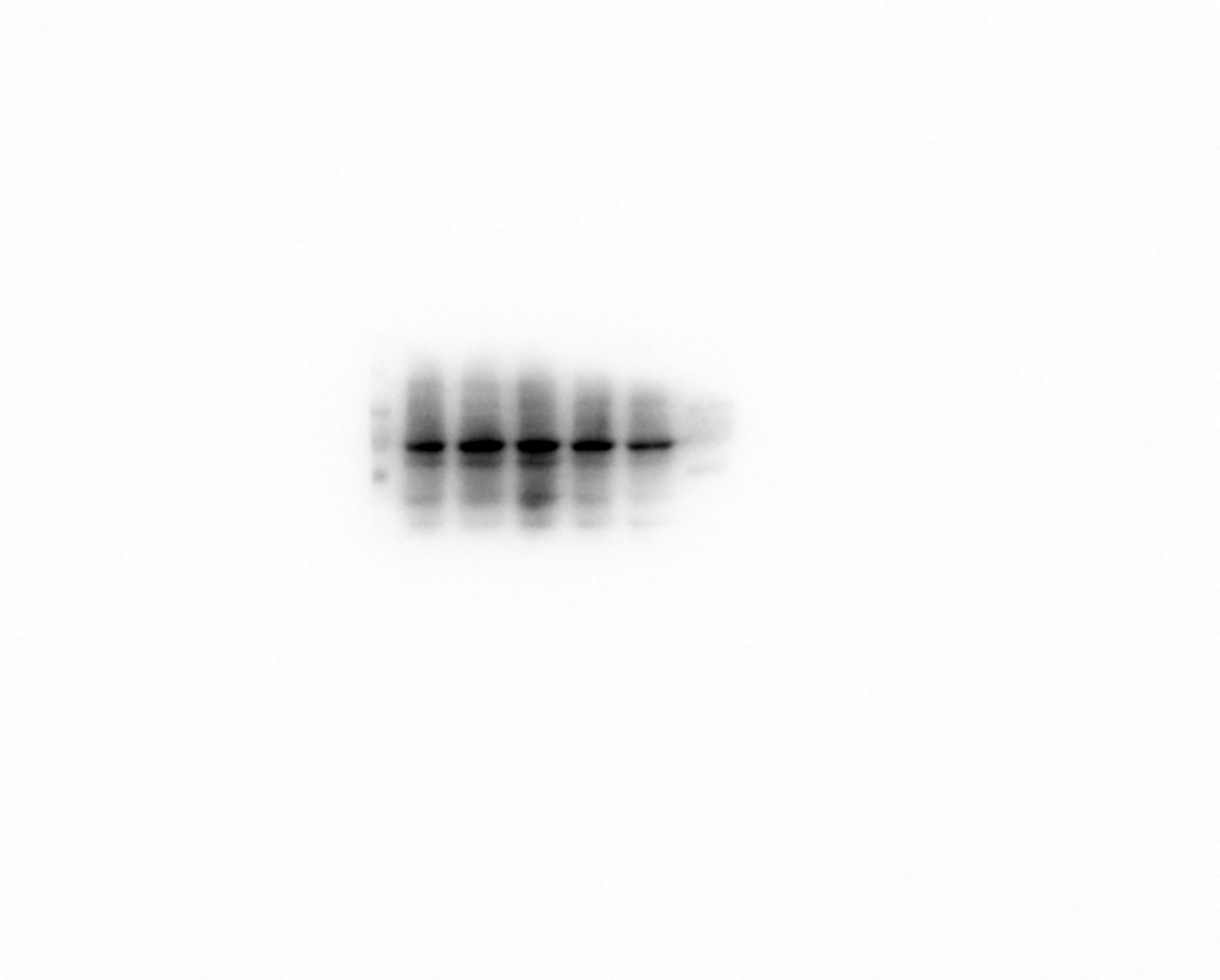

Supplement: Supplementary file 1 [file DataSheet1.ZIP › whole images of WB/P-P65.Tif]

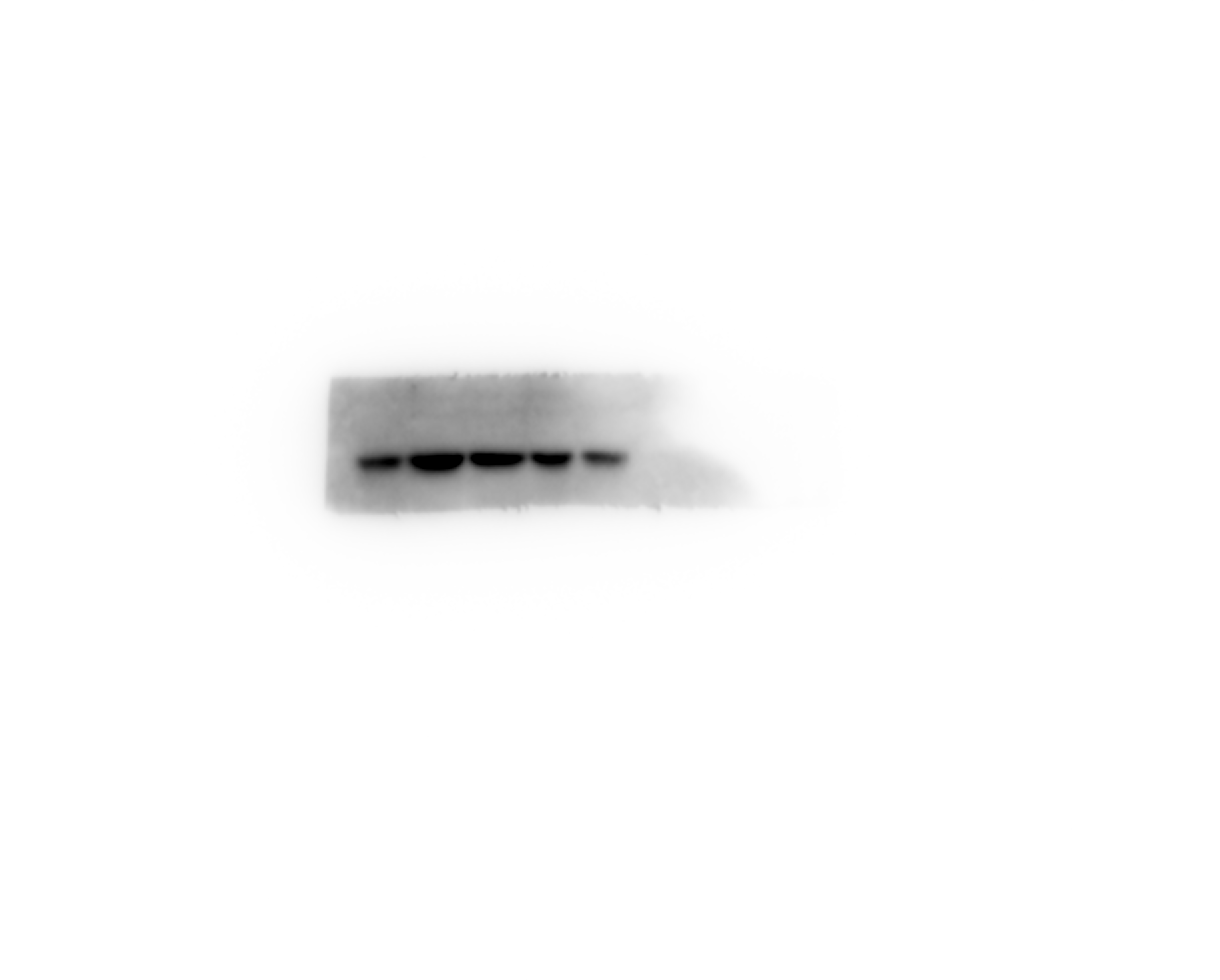

Supplement: Supplementary file 1 [file DataSheet1.ZIP › whole images of WB/TNF-a.Tif]

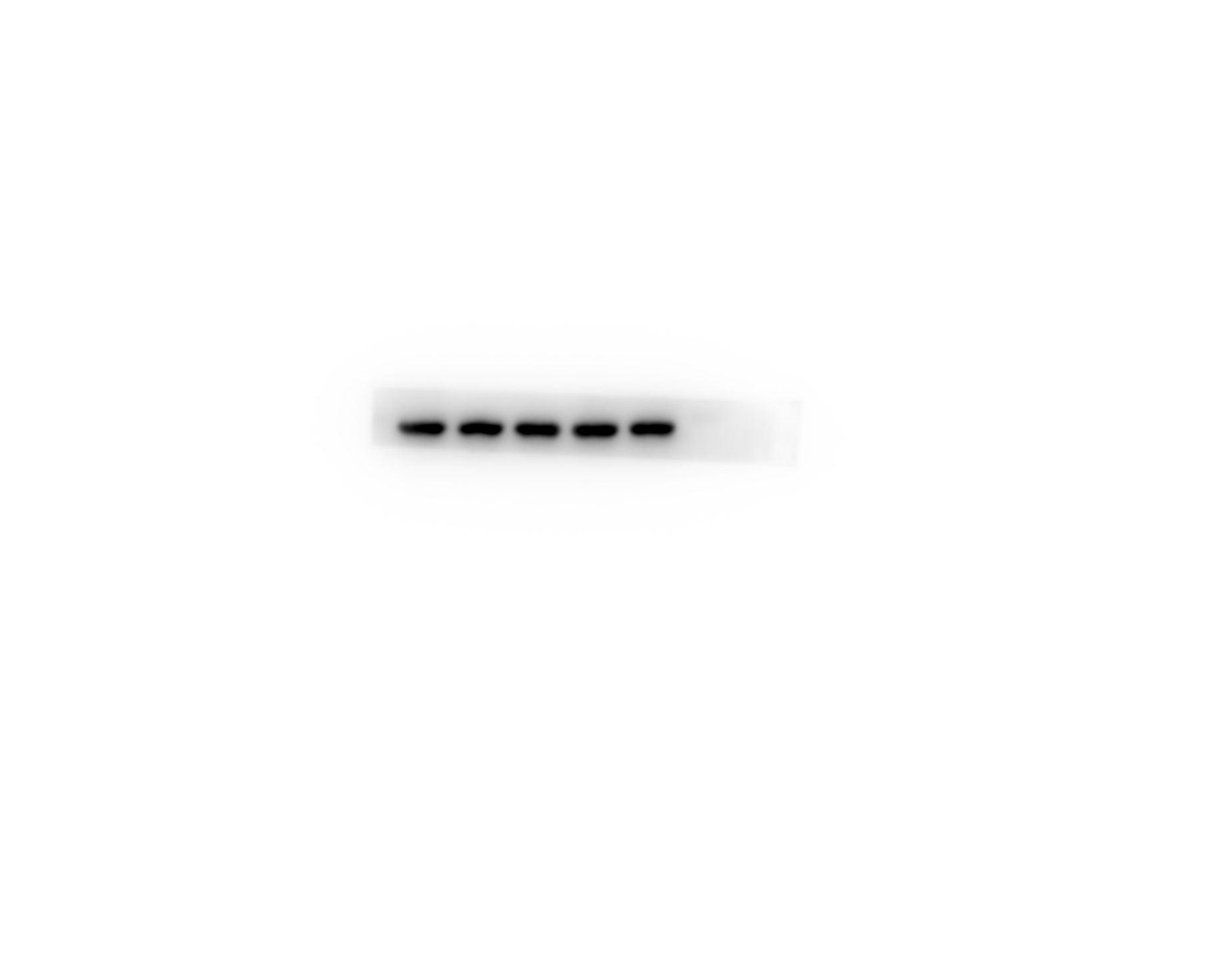

Supplement: Supplementary file 1 [file DataSheet1.ZIP › whole images of WB/b-Tubulin (nf-kb).Tif]

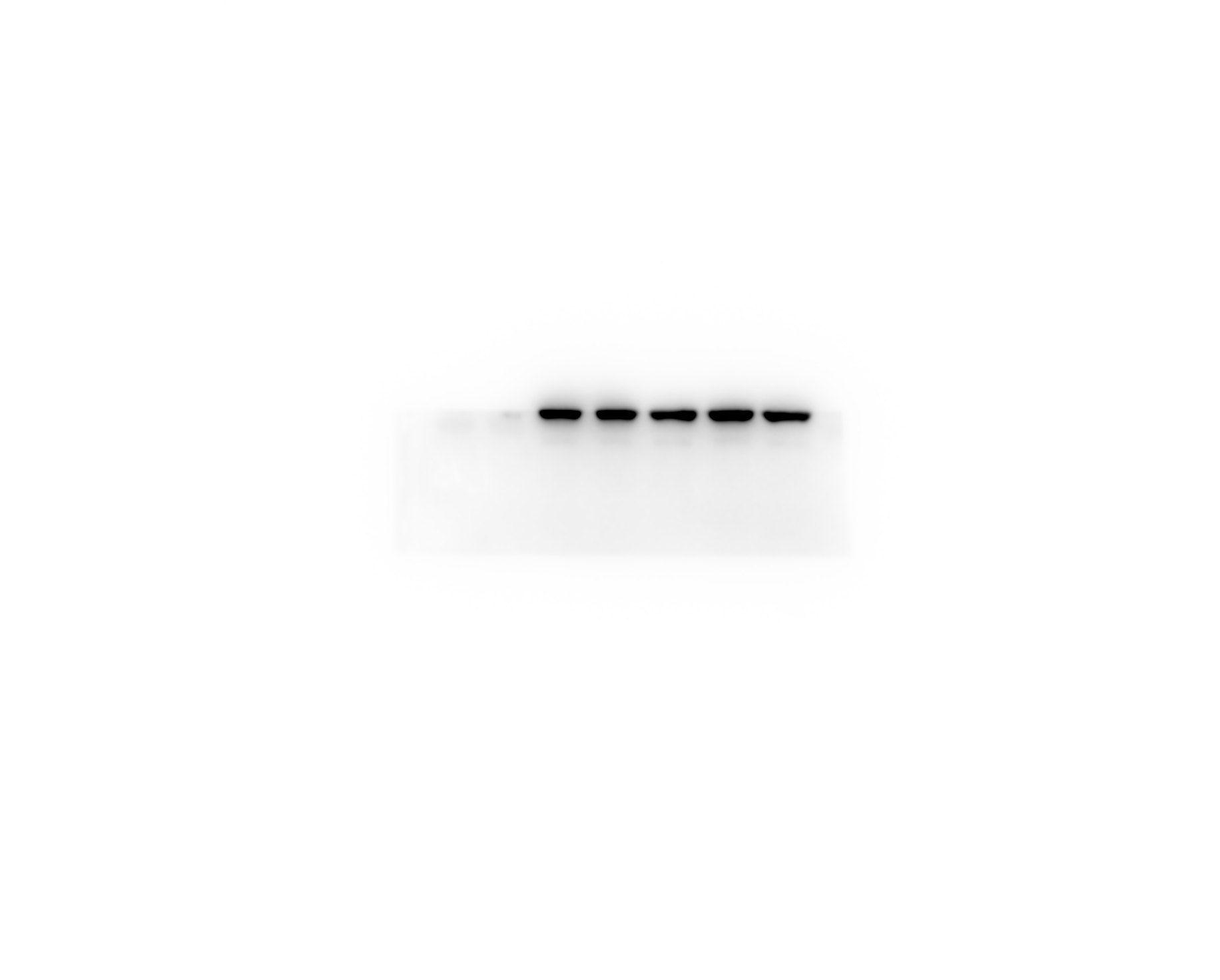

Supplement: Supplementary file 1 [file DataSheet1.ZIP › whole images of WB/b-Tubulin ú¿IL-1bú⌐.Tif]

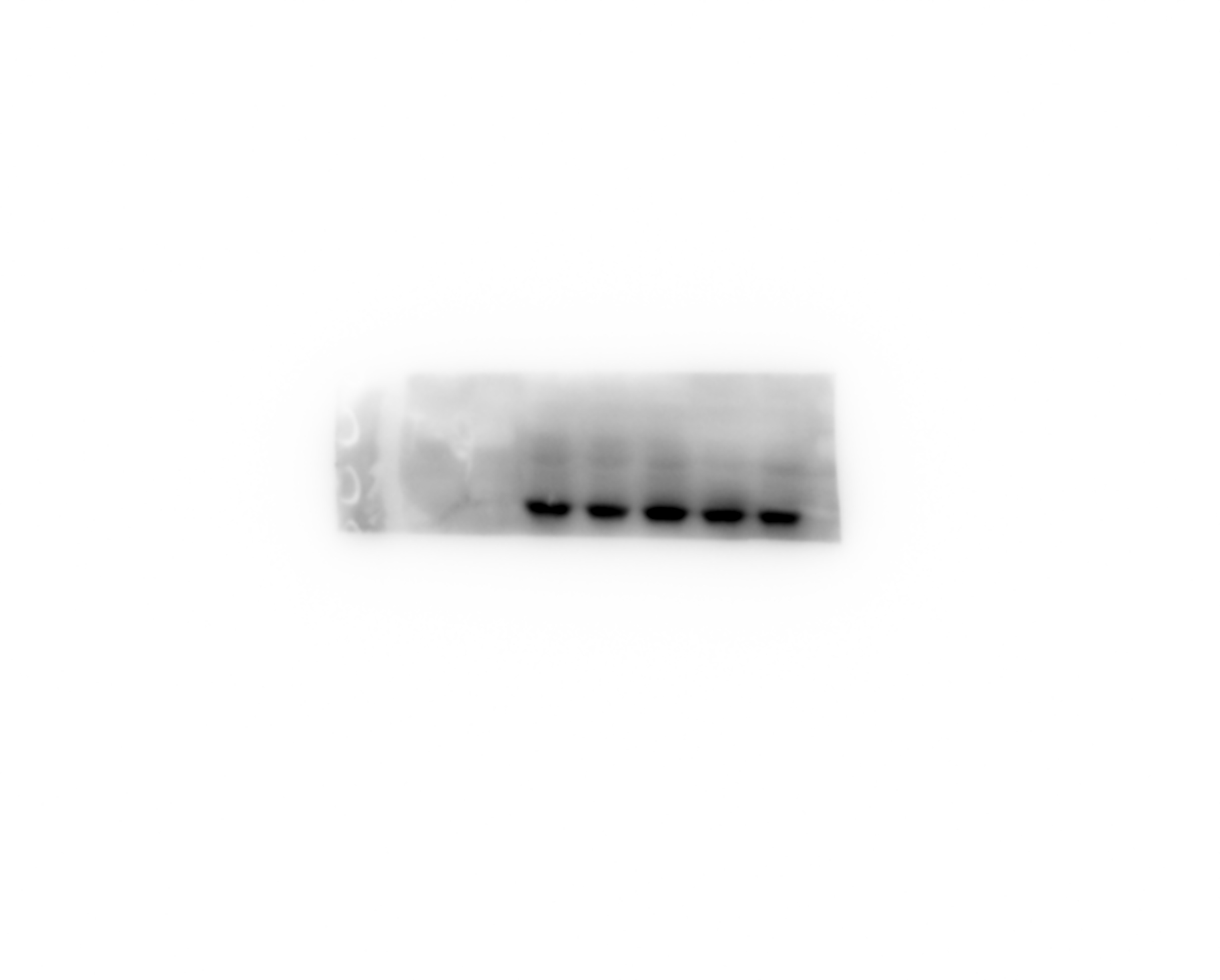

Supplement: Supplementary file 1 [file DataSheet1.ZIP › whole images of WB/b-Tubulin ú¿TNF-aú⌐.Tif]

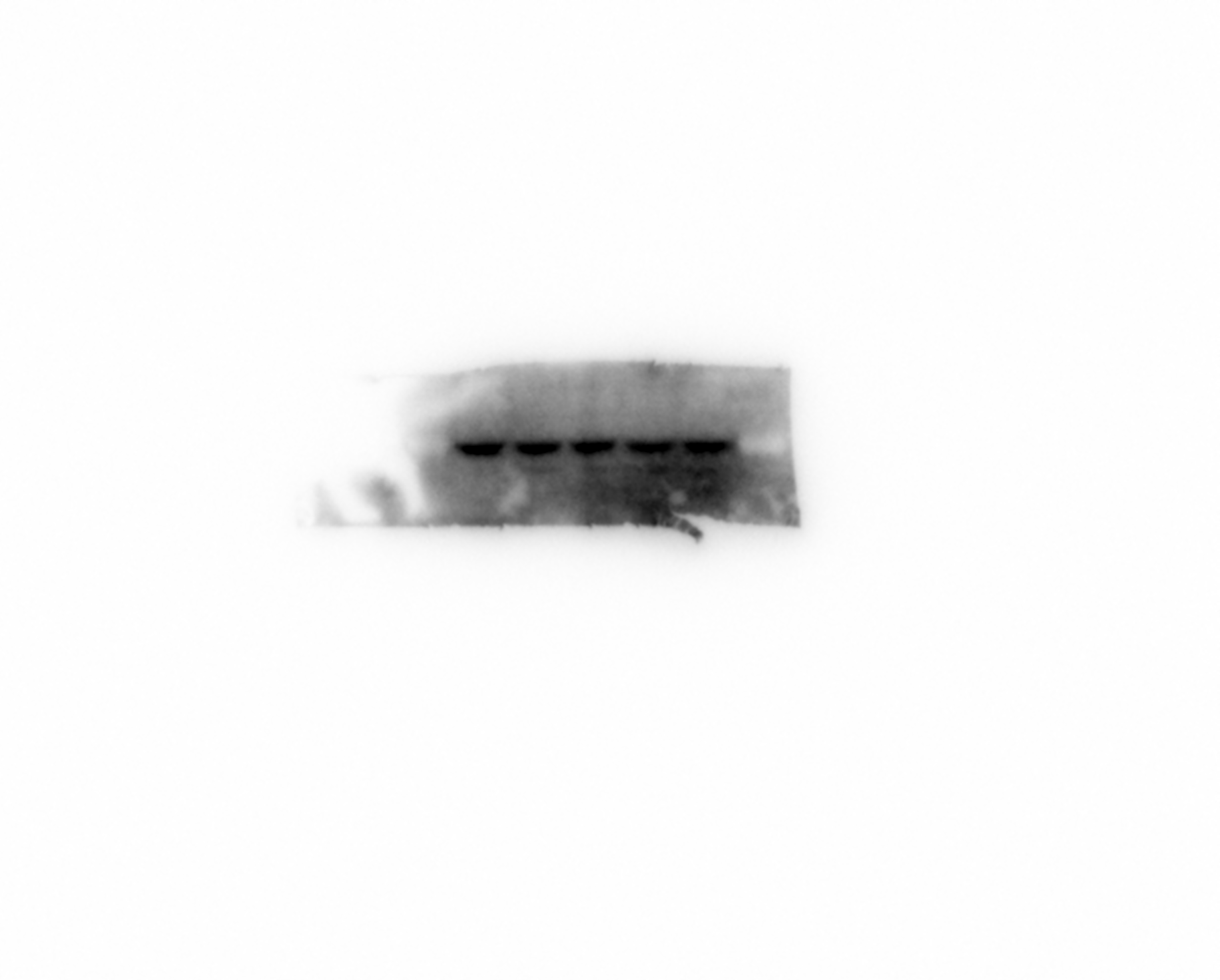

Supplement: Supplementary file 1 [file DataSheet1.ZIP › whole images of WB/lysate NF-KB p65.Tif]

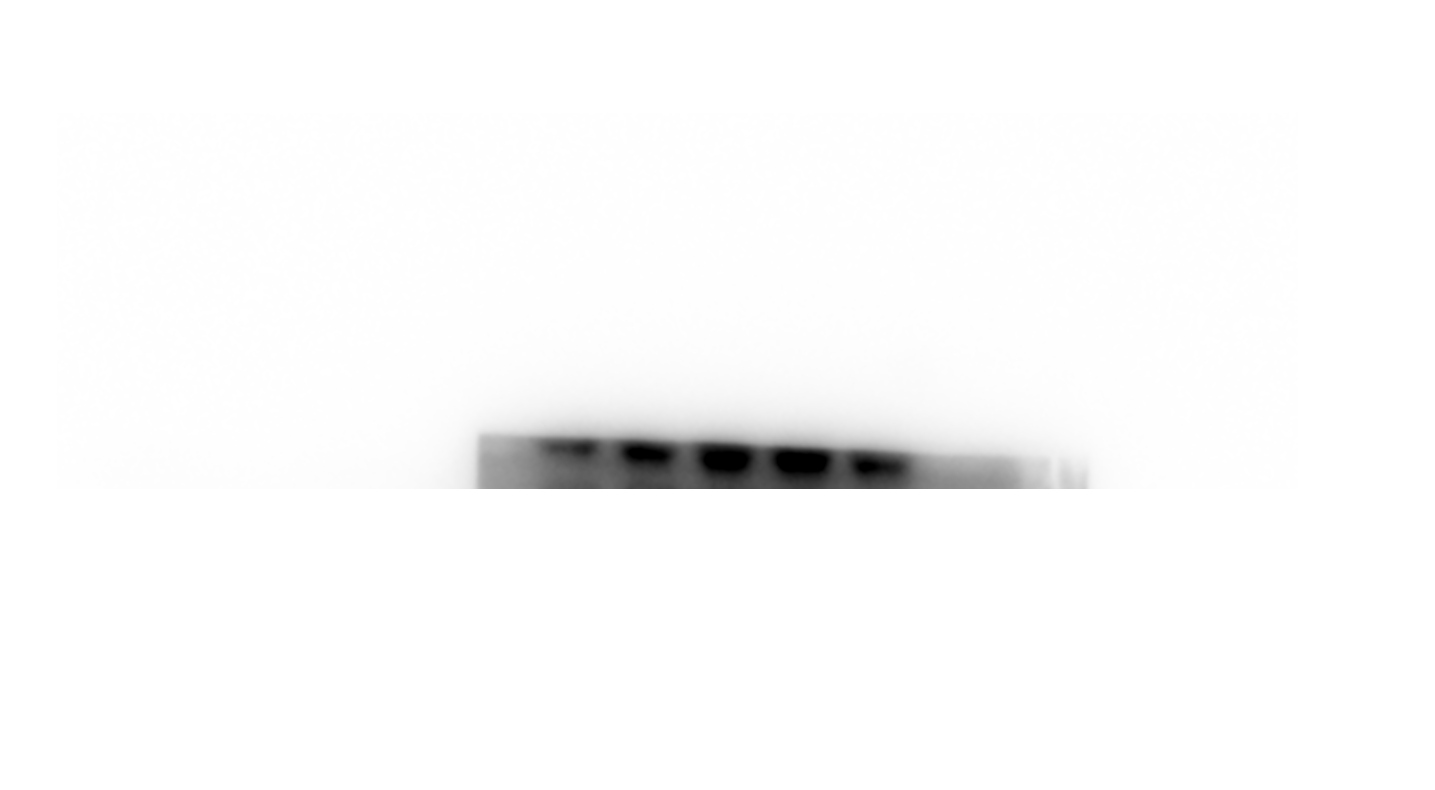

Supplement: Supplementary file 1 [file DataSheet1.ZIP › whole images of WB/mature IL-1b.tif]

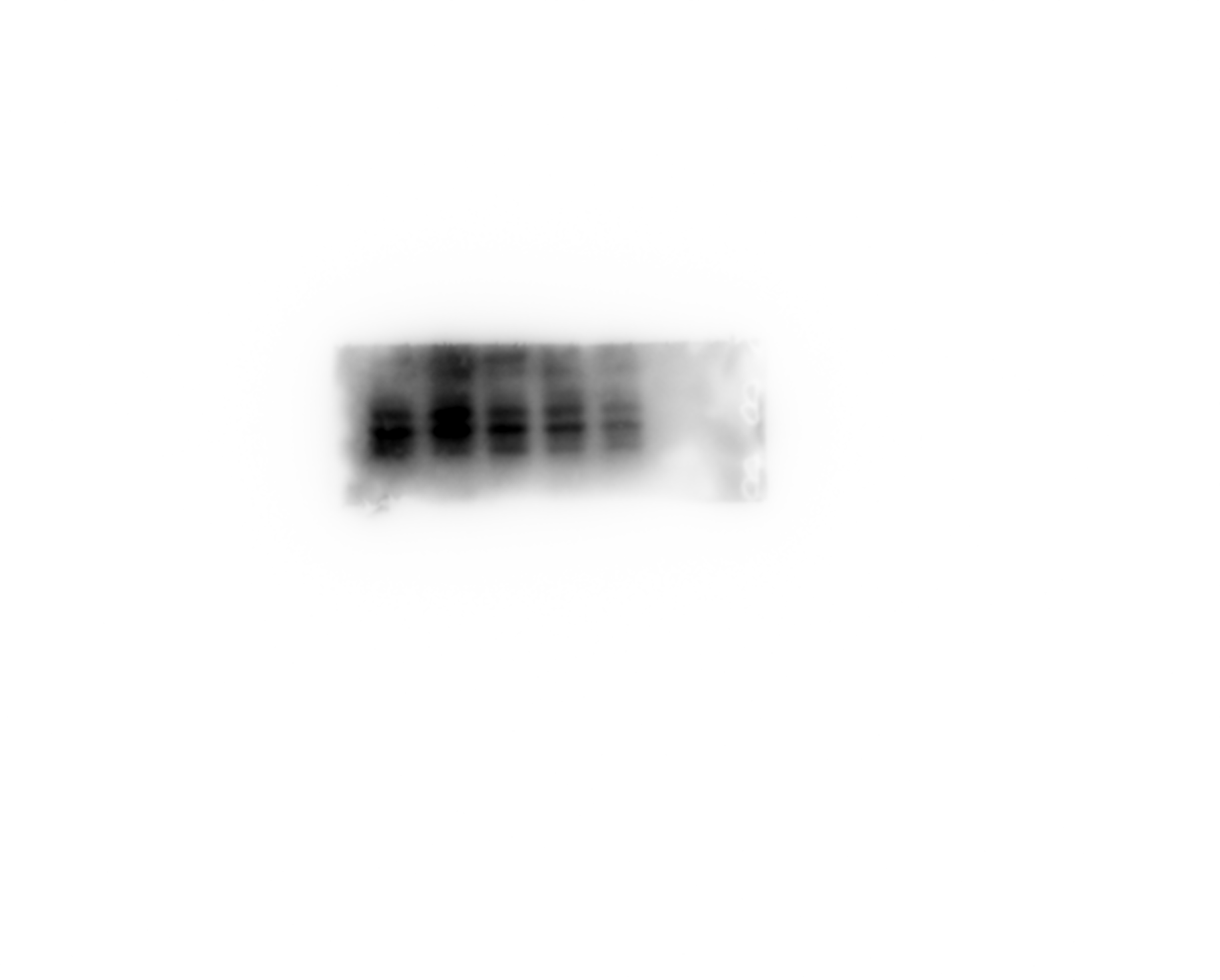

Supplement: Supplementary file 1 [file DataSheet1.ZIP › whole images of WB/nucleus NF-KB p65.Tif]

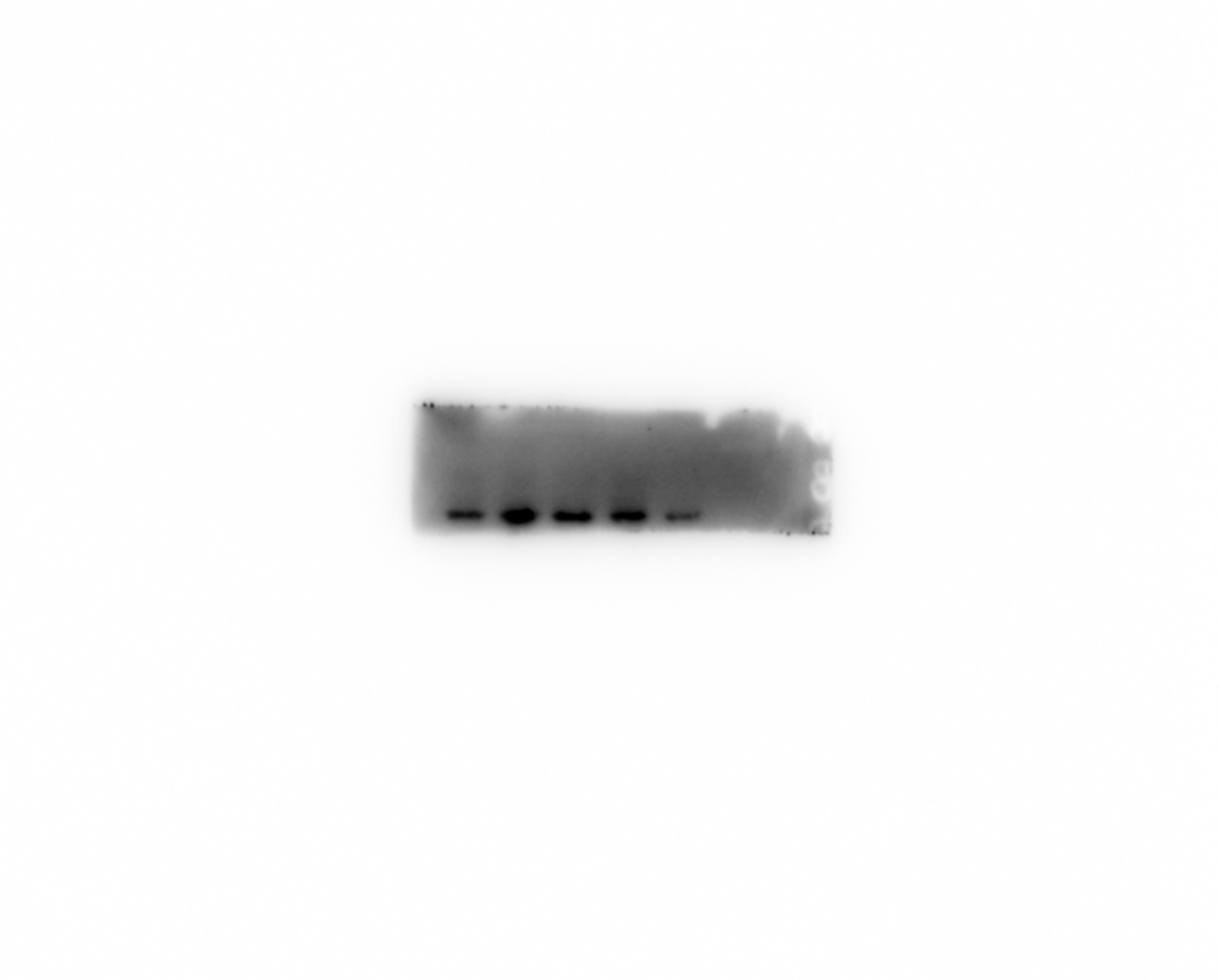

Supplement: Supplementary file 1 [file DataSheet1.ZIP › whole images of WB/p-IKBA.Tif]

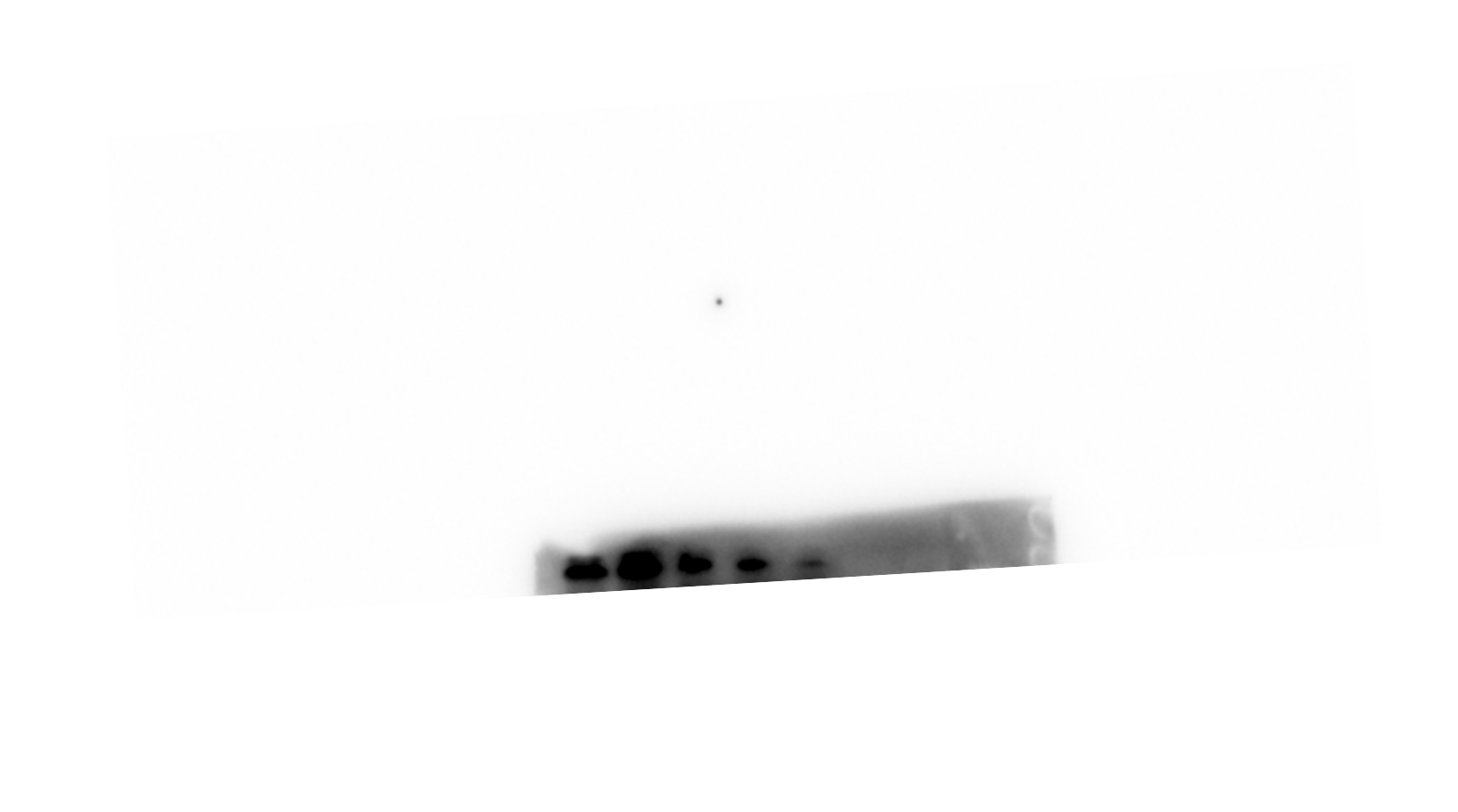

Supplement: Supplementary file 1 [file DataSheet1.ZIP › whole images of WB/p-IKK.tif]

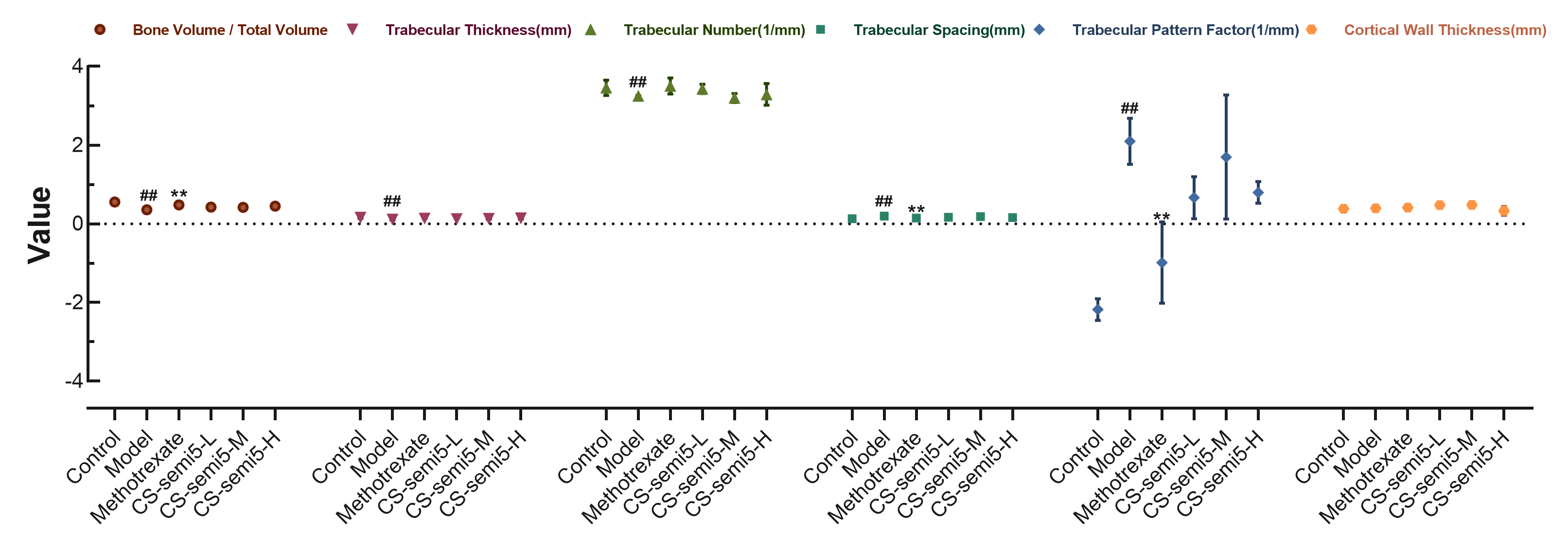

Supplement: Supplementary file 2 [file Image1.TIF]
